# *Legionella pneumophila* IrsA, a novel, iron-regulated exoprotein that facilitates growth in low-iron conditions and modulates biofilm formation

**DOI:** 10.1128/spectrum.02313-24

**Published:** 2024-11-29

**Authors:** Alberto E. Lopez, Joshua Mayoral, Huaixin Zheng, Nicholas P. Cianciotto

**Affiliations:** 1 Department of Microbiology and Immunology, Northwestern University Medical School, Chicago, Illinois, USA; McGill University, Quebec, Canada

**Keywords:** *Legionella pneumophila*, RNA-Seq, iron acquisition, FeoB, T2SS, type II secretion, biofilm, autoaggregation

## Abstract

To discover new factors that are involved in iron acquisition by *Legionella pneumophila*, we used RNA-Seq to identify the genes that are most highly induced when virulent strain 130b is cultured in a low-iron chemically defined medium. Among other things, this revealed *14915*, a heretofore uncharacterized gene that is predicted to be transcriptionally regulated by Fur and to encode a novel, ~15 kDa protein. *14915* was present in all *L. pneumophila* strains examined and had homologs in a subset of the other *Legionella* species. Compatible with it containing a classic signal sequence, the 14915 protein was detected in bacterial culture supernatants in a manner dependent upon the *L. pneumophila* type II secretion system. Thus, we designated 14915 as IrsA for iron-regulated, secreted protein A. Based on mutant analysis, the *irsA* gene was not required for optimal growth of strain 130b in low-iron media. However, after discovering that the commonly used laboratory-derived strain Lp02 has a much greater requirement for iron, we uncovered a growth-enhancing role for IrsA after examining an Lp02 mutant that lacked both IrsA and the Fe^2+^-transporter FeoB. The *irsA* mutant of 130b, but not its complemented derivative, did, however, display increased biofilm formation on both plastic and agar surfaces, and compatible with this, the mutant hyper-aggregated. Thus, IrsA is a novel, iron-regulated exoprotein that modulates biofilm formation and, under some circumstances, promotes growth in low-iron conditions. For this study, we determined and deposited in the database a complete and fully assembled genome sequence for strain 130b.

**IMPORTANCE:**

The bacterium *Legionella pneumophila* is the principal cause of Legionnaires’ disease, a potentially fatal form of pneumonia that is increasing in incidence. *L. pneumophila* exists in many natural and human-made water systems and can be transmitted to humans through inhalation of contaminated water droplets. *L. pneumophila* flourishes within its habitats by spreading planktonically, assembling into biofilms, and growing in larger host cells. Iron acquisition is a key determinant for *L. pneumophila* persistence in water and during infection. We previously demonstrated that *L. pneumophila* assimilates iron both by secreting a non-protein iron chelator (siderophore) and by importing iron through membrane transporters. In this study, we uncovered a novel, secreted protein that is highly iron-regulated, promotes *L. pneumophila*’s growth in low-iron media, and impacts biofilm formation. We also identified uncharacterized, IrsA-related proteins in other important human and animal pathogens. Thus, our results have important implications for understanding iron assimilation, biofilm formation, and pathogenesis.

## INTRODUCTION

The Gram-negative bacterium *Legionella pneumophila* is the main agent of Legionnaires’ disease, a form of pneumonia that is both increasing in incidence and especially problematic for immunocompromised and elderly individuals (1, 2). In natural and human-made water systems, *L. pneumophila* survives planktonically, as a constituent of biofilms and as an intracellular parasite of numerous types of amoebae (3, 4). Humans are primarily infected with *L. pneumophila* by the inhalation of contaminated water droplets that are produced by a variety of aerosol-generating devices (5). Pneumonia ensues following bacterial growth in alveolar macrophages and the onset of extensive inflammation and tissue destruction (6). In both protozoan and mammalian host cells, *L. pneumophila* replicates in a unique, membrane-bound compartment (Legionella-containing vacuole [LCV]), and this infectious process occurs, in large part, through the action of the Dot/Icm type IV protein secretion system (T4SS) (7). *L. pneumophila* also utilizes a type II protein secretion system (T2SS) to flourish extracellularly, form biofilms, and grow in host cells (8, 9). Iron acquisition is yet another determinant for *L. pneumophila* persistence in water systems, intracellular infection of host cells, and virulence (10, 11).

As one of its main modes of iron acquisition, *L. pneumophila* imports ferrous iron through the FeoB inner membrane transporter, which is encoded by the *feoABC* operon (12, 13). Indeed, *feoB* mutants are impaired for growth in low-iron media, biofilm formation, infection of amoebae and macrophages, and survival in the lungs of experimentally infected animals (12, 14). To facilitate its acquisition of Fe^2+^, *L. pneumophila* secretes homogentisic acid which polymerizes to form a melanin-like pigment that acts as an extracellular ferric reductase (15–17). Another facilitator of *L. pneumophila* ferrous iron acquisition and growth in low-iron conditions is the protein MavN/IroT, which, during intracellular infection, can be delivered into the membrane of the LCV by the Dot/Icm T4SS and therein serve as a transporter of Fe^2+^ and other cations (14, 18–21). As a second major pathway for iron uptake, *L. pneumophila* secretes a Fe^3+^-chelating siderophore known as rhizoferrin (22, 23). Detected when *L. pneumophila* is grown in a deferrated chemically defined medium, rhizoferrin is synthesized by the siderophore synthetase LbtA and then exported via the membrane protein LbtB (22, 24–27). Subsequently, ferri-rhizoferrin is imported by the combined action of the outer membrane receptor LbtU and the inner membrane transporter LbtC (28, 29). From phenotypic analyses of an *lbtA* mutant and an *lbtA feoB* mutant, we determined that rhizoferrin is necessary for the optimum growth of *L. pneumophila* in low-iron media, in host cells, and in the lungs of mice (14, 28–30). Additionally, rhizoferrin, like FeoB-mediated ferrous transport, can promote biofilm formation by *L. pneumophila* (14). *L. pneumophila* regulates the expression of its *lbtABC*, *lbtU, feoABC,* and *mavN/iroT* genes through the action of Fur, which is a transcriptional repressor when iron levels are sufficiently elevated and is itself modulated by CsrA (12, 18, 19, 28, 29, 31–34). The growth of *L. pneumophila* in low-iron conditions and the production of siderophore activity by the bacterium are also promoted by cytochrome c_4_ and the cytochrome *c* maturation (*ccm*) locus, although the manner in which this occurs remains largely unknown (10, 35–37). As is the case for many other bacteria, *L. pneumophila* has yet additional factors that are induced by low-iron conditions and/or promote growth in iron-deplete media or during intracellular infection (10). Among these factors are (i) a putative second siderophore synthetase that is Fur-regulated, shares sequence similarly with LbtA, and promotes macrophage infection (FrgA), (ii) a paralog of LbtU that can aid in ferri-siderophore uptake (LbtP), (iii) a putative membrane-associated, peptide transporter that fosters growth in low-iron media (IraB), (iv) hemin utilization and a surface hemin-binding protein, (v) a T2SS-dependent, secreted metalloprotease that can degrade the mammalian iron-binding protein transferrin (ProA), (vi) the twin-arginine translocon, which enhances growth in low-iron conditions presumably due to its role in protein secretion (TatB), (vii) a periplasmic, multi-copper oxidase that aids growth in the presence of Fe^3+^ sources (McoL), (viii) cell-associated ferric reductases that may reduce imported ferric iron or ferri-siderophore (Cfr, Pfr), (ix) an iron-regulated, mini-ferritin-like protein (DpsL), and (x) homologs of pyoverdine synthesis genes that are iron-regulated and hyperexpressed in biofilms (PvcA, PvcB) (10, 26, 29, 32, 38–48). In the current study, we have uncovered a novel, highly iron-regulated protein (to be designated as IrsA, for iron-regulated, secreted protein A) that is secreted by the T2SS and impacts *L. pneumophila*’s ability to grow in low-iron conditions and to form biofilm.

## RESULTS

### RNA-Seq analysis of *L. pneumophila* strain 130b grown in a low-iron, chemically defined medium

**Fig 1:**
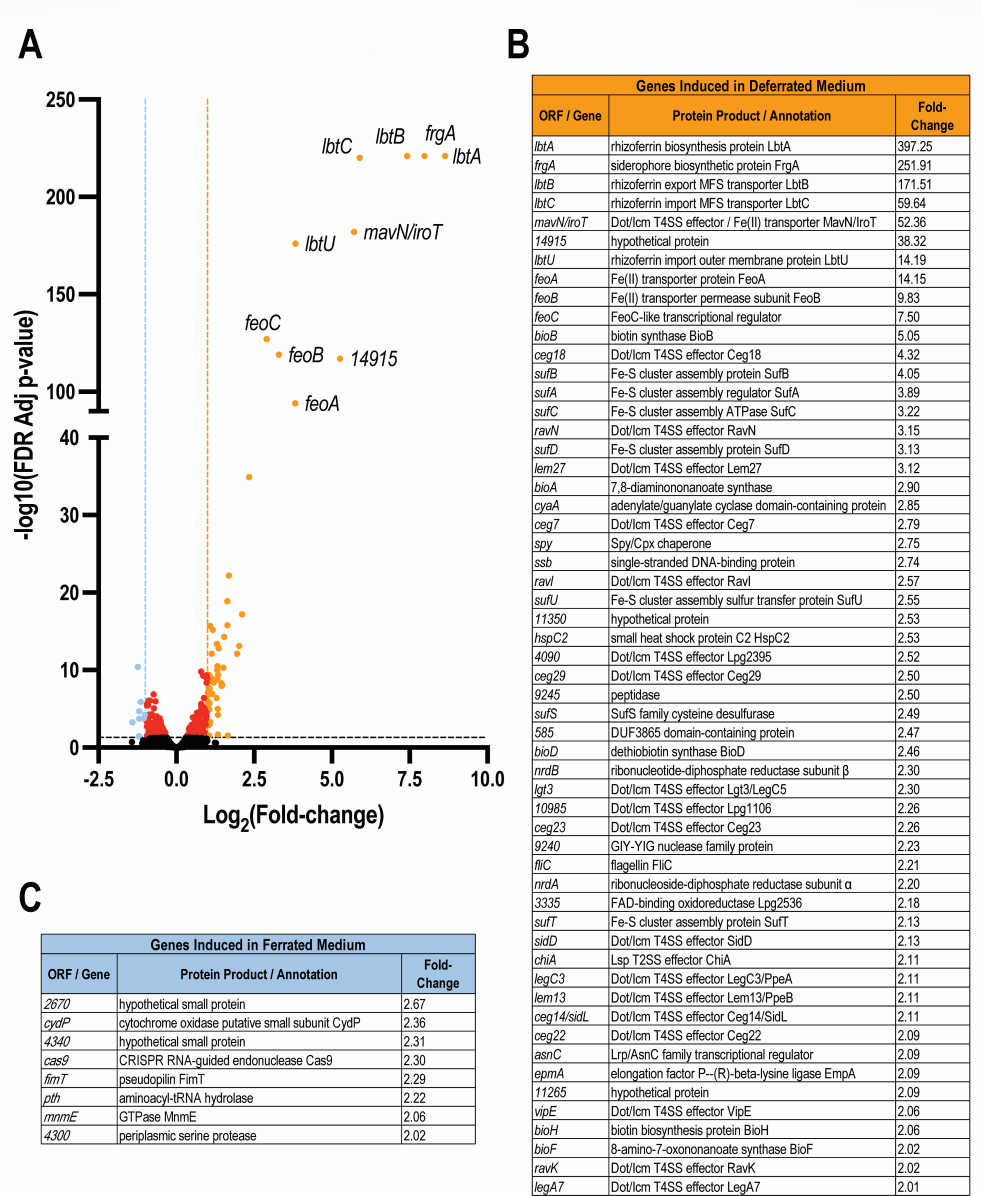
Identification of *L. pneumophila* genes that are induced by bacterial growth in deferrated CDM. On three independent occasions, *L. pneumophila* wild-type strain 130b was grown to mid-log phase in either CDM lacking any added iron (CDM – Fe) or CDM containing 5 µM added ferric pyrophosphate (CDM + Fe), and then RNAs isolated from the three biological replicates per growth condition were subjected to RNA-Seq analysis. (**A**) Volcano plot depicting the response of *L. pneumophila* genes to bacterial growth in CDM – Fe vs growth in CDM + Fe. Orange dots to the right of the vertical orange line denote the genes that were more highly expressed by at least twofold (*P* < 0.05) during growth in CDM – Fe. Blue dots to the left of the vertical blue line denote the genes that were more highly expressed by at least twofold (*P* < 0.05) during growth in CDM + Fe. Red dots indicate those genes that were more modestly affected by changes in iron, and black dots signify genes whose expression was not impacted at all by iron levels. The names or ORF designation for the 10 genes that were most highly induced by iron depletion are indicated in the upper right portion of the plot. (**B**) Table presenting the ORF designation/gene name, protein product/annotation, and fold induction (*P* < 0.05) for the 56 genes that were induced by growth in deferrated medium. (**C**) Table showing the ORF designation/gene name, protein product/annotation, and fold induction (*P* < 0.05) for the eight genes that were induced by growth in ferrated medium. (A–C) As per NCBI, the names for all the ORFs in the complete 130b genome formally consist of the prefix “ABXK18_” followed by a three- to five-digit number, e.g., “ABXK18_XXXXX.” For simplicity, the graph and tables provide only the numerical part of the ORF designation.

To uncover “new” factors that might be linked to rhizoferrin or other aspects of *L. pneumophila* iron acquisition, we performed a transcriptomic analysis of *L. pneumophila* grown in media containing different levels of iron. Specifically, we cultured wild-type (WT) strain 130b, a clinical isolate belonging to serogroup-1, in chemically defined medium (CDM) that lacks any iron added to the base medium (CDM – Fe) vs in CDM that includes an iron supplement of 5 µM ferric pyrophosphate (CDM + Fe) and then using RNA-Seq analysis identified those transcripts that are more highly expressed in the iron-deplete cultures. The CDM base consists of the 20 amino acids (aas) (with cystine replacing cysteine), α-ketoglutarate, glutathione, pyruvate, nine trace metals other than iron, KH_2_PO_4_, NaCl, and MOPS buffer. As alluded to earlier, CDM − Fe is the medium that has been used for the detection and purification of *L. pneumophila* rhizoferrin (22–25). Utilizing inductively coupled plasma mass spectrometry (ICP-MS), we determined that the level of iron in our CDM – Fe was equal to 0.14 µM. There has been only one prior effort at using transcriptomics to identify iron-regulated genes of *L. pneumophila*, and that study used microarrays to find RNAs that are more highly expressed when strain Paris is grown for 30 min or 3 h in a complex medium (i.e., buffered yeast extract, BYE) that was supplemented with an iron chelator (i.e., 20 µM deferoxamine, DFX) (18). As a prelude to our transcriptomic analysis, we determined the complete and fully assembled genome sequence for *L. pneumophila* strain 130b, since a previous genomic study had only reported a draft sequence for that strain (49). For our investigation, we employed CDM – Fe cultures and CMD + Fe cultures that had been grown for ~4 h to mid-log phase, since quantitative reverse-transcription polymerase chain reaction (qRT-PCR) analysis determined that the induction of known iron acquisition genes (e.g., *lbtA* and *lbtU*) is greatest during the mid-log phase as compared to the late-log and early-stationary phases (Fig. S1). In total, the RNA-Seq analysis uncovered 56 genes that were notably more expressed (≥2-fold) in the bacteria grown in CDM – Fe as compared to the bacteria grown to a similar extent in CDM + Fe, i.e., genes induced by iron depletion (Fig. 1A and B). This was in addition to finding eight genes that were notably more expressed (≥2-fold) in those samples grown in CDM + Fe, i.e., genes repressed upon iron depletion (Fig. 1A and C). Table S1 contains the full data set of all RNAs detected in the samples. We gave our immediate attention to the 10 genes that were most induced upon iron depletion (Fig. 1A and B). Not surprisingly, four of the genes that were most induced in the low-iron condition were the genes known to be involved in rhizoferrin synthesis (*lbtA*), export (*lbtB*), or import (*lbtC, lbtU*) (26, 28, 29). The *lbtA* gene stood out as being the most hyperexpressed showing a 397-fold induction in low iron, followed by *lbtB* at 172-fold induction, *lbtC* at 60-fold induction, and *lbtU* at 14-fold induction. Incidentally, although *lbtA*, *lbtB*, and *lbtC* are known to exist in an operon (that is separate from *lbtU*) (28, 29), the variation in their induction levels most likely reflects degradation of transcripts from their 3´ ends. The second-most induced gene overall (at 252-fold) was *frgA*, the previously defined Fur-regulated gene that promotes intracellular infection and encodes a protein homologous to LbtA (26, 32). The fifth-most induced gene (at 52-fold) was *mavN/iroT*, another previously described iron-regulated gene that promotes growth in low-iron media and during intracellular infection (18, 19). Also expectedly, the *feoA, feoB*, and *feoC* genes that are linked to ferrous iron transport (12) were found to be induced 7- to 14-fold upon growth in CDM − Fe. All of the aforementioned nine genes had been detected in the microarray analysis of strain Paris grown in DFX-containing BYE broth (18). Other iron-related genes that were induced (~3- to 4-fold) by low-iron conditions in both our examination and the prior microarray study were eight genes in an operon beginning with *sufA* that promote the incorporation of iron-sulfur clusters into select proteins (Fig. 1B) (18, 43, 50, 51). However, our analysis, most importantly, uncovered a gene, i.e., open reading frame (ORF) “*14915*,” that had not been previously reported and was induced 38-fold in the low-iron growth condition (Fig. 1A and B). Given its novelty, *14915* became the focus of our attention.

### Transcription of the *14915* gene is highly responsive to iron levels

**Fig 2:**
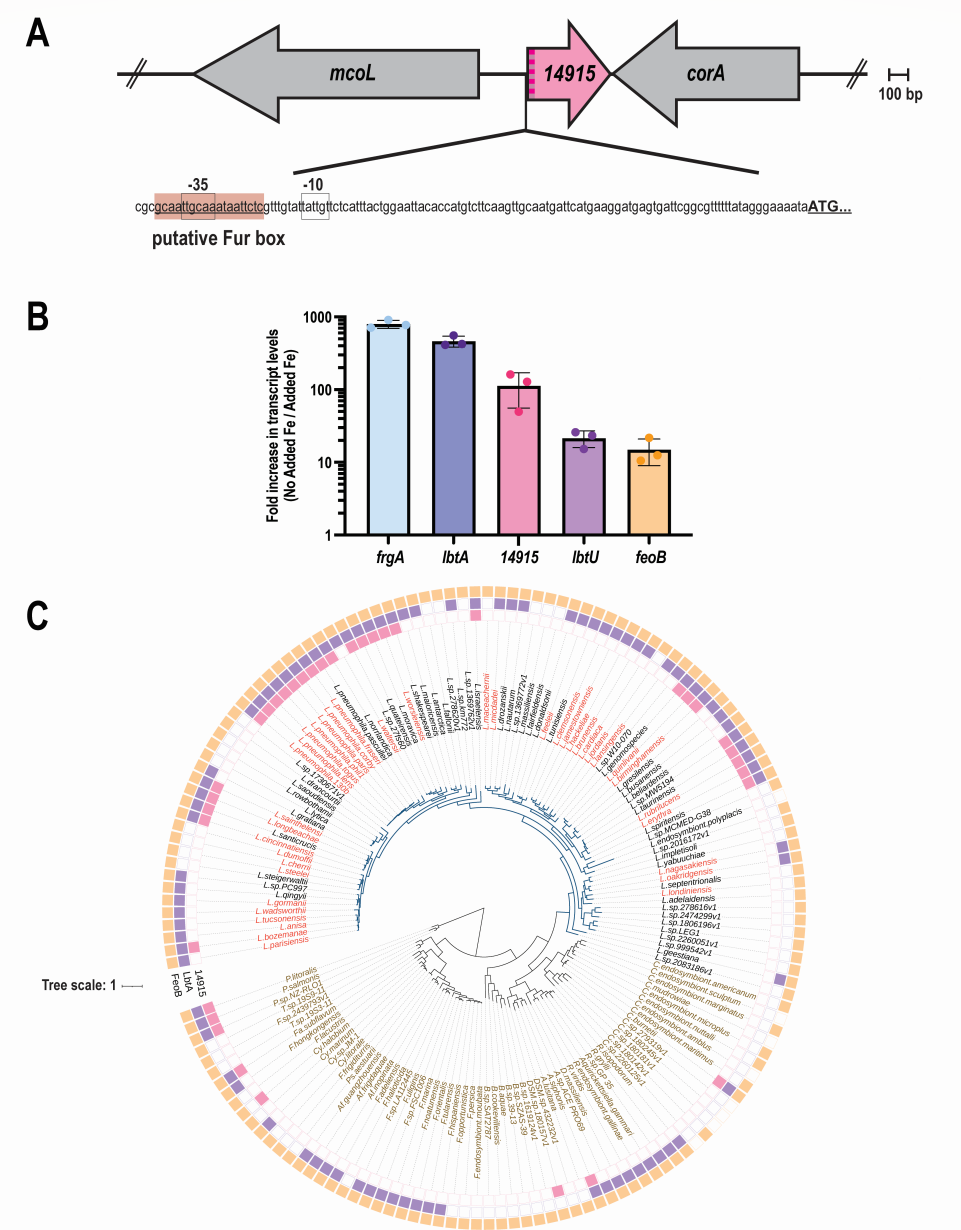
Genetic locus, iron-regulated transcription, and phylogenetic distribution of the *14915* gene. (**A**) Depiction of the chromosomal locus of *L. pneumophila* strain 130b that encodes *14915*. In the gene map presented at the top, the arrows indicate the relative length of each gene and the direction of their transcription. The lower line is the nucleotide sequence upstream of the *14915* ORF, showing its predicted promoter region (i.e., −10 box and −35 box, spaced 17 bp apart) and the putative binding site for the Fur repressor. (**B**) Effect of low-iron growth conditions on the induction of *14915* expression. WT strain 130b was grown to mid-log phase in either CDM lacking added iron (CDM − Fe) or CDM containing 5 µM added ferric pyrophosphate (CDM + Fe), and then mRNA levels for each of the five indicated genes were determined by qRT-PCR using, as the templates isolated, whole-cell RNA from three biological replicates (examined in triplicate). Presented are the means and standard deviations for the fold increases in transcript levels when comparing samples from CDM − Fe to those from CDM + Fe. (**C**) Distribution of genes encoding 14915/IrsA homologs within species of *Legionella* and related genera. (Center) A maximum-likelihood phylogenetic tree for unique species in the orders *Berkiellales*, *Coxiellales*, *Diplorickettsiales*, *DSM-16500*, *Francisellales, Legionellales*, and *Piscirickettsiales*. Scale, one amino acid substitution per site. *Legionella* species colored in red have been associated with human disease, and those in black have not (yet) been linked to disease. Non-*Legionella* species that belong to other genera are colored in brown. (Outer rings) The presence of a gene corresponding to *14915*/*irsA* (pink squares), *lbtA* (purple), or *feoB* (gold) absence (white) are indicated, as determined using BLASTP.

Examination of the *L. pneumophila* 130b genome indicated that *14915* is a monocistronic gene that is predicted to encode a 144-amino-acid protein (Fig. 2A). Upstream of *14915* was *mcoL*, which, as noted in the Introduction, encodes a multi-copper oxidase that can promote *L. pneumophila* growth in the presence of ferric iron sources (44, 52). Downstream of *14915* was a gene annotated as *corA* and predicted to encode a magnesium/cobalt uptake transporter (53, 54). To verify our RNA-Seq results, we utilized qRT-PCR to further compare the levels of *14915* transcripts in CDM – Fe cultures vs CDM + Fe cultures. This experiment confirmed that *14915* is highly induced (~100-fold) by iron depletion, showing a level of induction that was greater than that of *lbtU* and *feoB* but less than that of *lbtA* and *frgA* (Fig. 2B), a result that was in-line with the RNA-Seq data. Further examination of the strain 130b genome revealed that the promoter region for *14915* has a putative binding site for Fur (“Fur box”) (Fig. 2A). Figure S2 presents an alignment of the predicted Fur box for *14915* with the previously predicted Fur boxes for *lbtABC, lbtU, frgA, mavN/iroT*, and *feoABC* (18), i.e., the other genes that were most highly induced by iron depletion (Fig. 1B). Thus, *14915* transcription is likely repressed by Fur during *L. pneumophila* growth in high-iron conditions but de-repressed and greatly expressed when the bacterium is in low-iron environments.

### The *14915* gene is uniquely distributed in the *Legionellales*

BLASTP analysis identified genes/putative proteins corresponding to *14915*/14915 in 56 out of 56 other sequenced strains of *L. pneumophila* examined (Table S2), including the frequently studied strains Philadelphia-1 (i.e., ORF *lpg0266*), Paris (*lpp0340*), Lens (*lpl0318*), and Corby (*lpc0342*) (55–57). The average aa identity of the proteins in the different strains to the 14915 protein of strain 130b was 94.27%. The level of gene/protein conservation implied that 14915 and its homologs serve an important role for *L. pneumophila*. Incidentally, for the other strains examined, there was synteny at the *14915* locus (Fig. 2A) and strong sequence conservation for the predicted McoL and CorA proteins (Table S2). Based on further BLASTP results, proteins closely related to 14915 were also encoded by strains representing 20 of the other sequenced species of *Legionella* (Fig. 2C). The level of relatedness to 14915 ranged from 72% aa identity (*E* = 8e − 73) to a protein in *Legionella norrlandica* to 47% aa identity (*E* = 1e − 42) to a protein in *Legionella bozemanae*. The distribution of the *14915*-like genes across the *Legionella* genus was notably different from that of other genes that were substantially induced upon iron depletion, including *lbtA* and *feoB* (Fig. 2C) as well as *lbtB*, *lbtC*, *lbtU,* and *mavN/iroT* (14), suggesting that *14915* might not be involved in the same aspect of iron acquisition or metabolism as these other factors. Hypothetical proteins related to 14915 were also encoded by strains representing a handful of other species in the *Legionellales* and related orders, including *Aquicella siphonis* (*E* = 3e − 32, 43% aa identity)*, Coxiella burnetii* (*E* = 2e − 19, 39% aa identity), *Piscirickettsia salmonis* (*E* = 3e − 17, 34% aa identity), *Facilibium subflavum,* (*E* = 3e − 12, 34% aa identity), *Cysteiniphilum halobium* (*E* = 3e − 12, 32% aa identity), and *Piscirickettsia litoralis* (*E* = 3e − 12, 38% aa identity) (Fig. 2C). Finally, predicted proteins with similarity to 14915 were detected in a strain of *Vibrio sinus* (*E* = 3e − 15, 34% aa identity), several species of dinoflagellates belonging to the *Symbiodinium* genus (e.g., *E* = 2e − 19, 33% aa identity for *Symbiodinium microadriaticum*)*,* and two species of rotifers in *Adineta* (e.g., *E* = 2e − 14, 35% aa identity for *Adineta ricciae*). Overall, BLASTP searches did not reveal significant similarity between 14915 and any known or characterized protein. Thus, we used AlphaFold 3 (58) to predict the 3-D structure of 14915. When this model (Fig. S3) was submitted to the DALI server (59), it did not yield significant matches to any known structures. Taken together, these data suggested that 14915 is representative of a heretofore uncharacterized group of proteins and therefore might possess a novel function.

### 14915 is a secreted protein and substrate of the *L. pneumophila* T2SS

**Fig 3:**
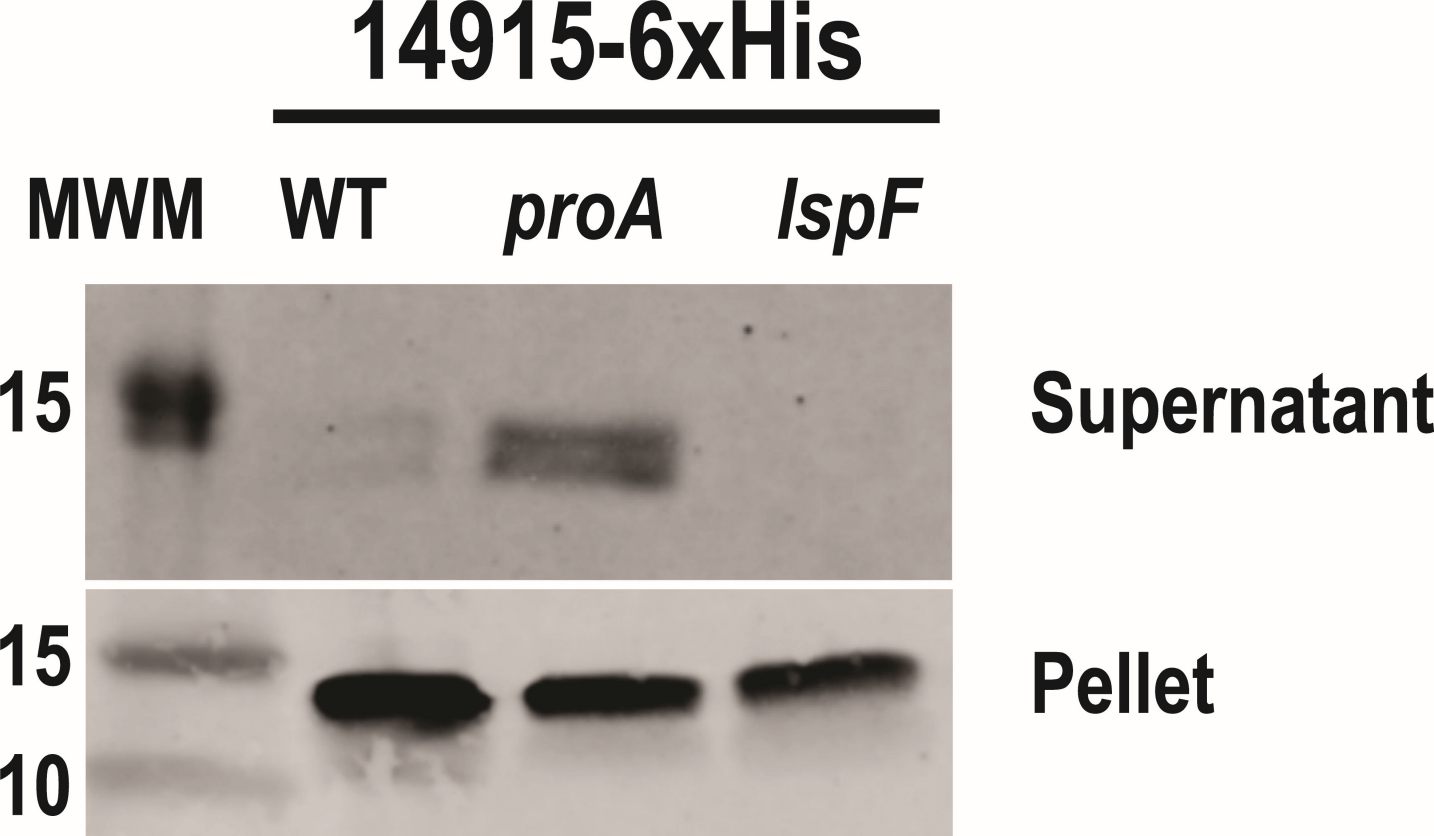
Detection of 14915 protein in culture supernatants of *L. pneumophila* strains and the effect of the T2SS on that protein secretion. Wild-type strain 130b (WT), *proA* mutant AA200 (*proA*), and *lspF* mutant NU275 (*lspF*) each containing an isopropyl ß-D-1-thiogalactopyranoside (IPTG)-inducible *14915* gene that encodes 14915 with a C-terminal 6xHis tag were inoculated into BYE broth and incubated for 1 h of protein induction. Cell-free culture supernatants concentrated 20-fold were subjected to 15% SDS-PAGE and then immunoblotted using anti-His antibodies (top panel). Cell pellets obtained from the same cultures were subjected to 4%–20% SDS-PAGE and the same immunoblot analysis (bottom panel). The left-most lane contained pre-stained molecular weight markers (MWM), whose sizes (in kDa) are noted. Presented here are the (only) portions of the blots showing proteins recognized by the antibodies, i.e., the 14915–6xHis protein. These results are representative of the outcome of at least three independent experiments, which also included 2 and 3 h of IPTG induction.

Based on SignalP analysis, 14915 has a 19-aa signal sequence at its N-terminus and therefore is undoubtedly transported out of the cytoplasm and into the periplasm by the Sec translocon. PSORTb analysis further suggested a subsequent extracellular localization for 14915. As noted in the Introduction, *L. pneumophila* expresses a T2SS, an envelope-spanning apparatus that transports proteins destined to be secreted from the periplasm into the extracellular milieu (8, 9). Thus, compatible with the fact that prior proteomic analyses of *L. pneumophila* culture supernatants had only examined legionellae grown in rich, iron-replete media, e.g., standard BYE broth (60–63), we posited that 14915 is a heretofore uncharacterized secreted protein and substrate of the T2SS. To test this hypothesis, we engineered a C-terminal 6-histidine (6xHis)-tagged version of 14915, introduced the construct into WT strain 130b, and then examined culture supernatants for the presence of 14915 using anti-His antibodies and immunoblotting, analogously to what we and others have done to determine the T2SS dependency of exoproteins in various other bacteria (64–67). Initially, not much of the protein was observed in the WT supernatants (Fig. 3, top panel). We postulated that 14915 and/or its tag were cleaved/degraded by the T2SS-dependent metalloprotease ProA, which is the major secreted protein in BYE broth cultures of *L. pneumophila* and is well known to cleave/degrade a range of other secreted proteins (8, 68). Indeed, when we examined supernatants from a *proA* mutant of strain 130b (69), the 14915–6xHis protein was easily seen on the immunoblot, migrating at its predicted size (Fig. 3, top panel). That the antibodies also recognized a slightly smaller form of protein suggested that 14915 might be cleaved after it is secreted into the supernatant. The protein was not detected when we examined culture supernatants of a *lspF* mutant that completely lacks the T2SS (70) (Fig. 3, top panel), strongly suggesting that 14915 is secreted via the T2SS, i.e., if it was secreted by a means other than the T2SS, then it would have been seen in the *lspF* mutant supernatants since they lack ProA. When we examined cell pellets derived from these same samples, all strains showed similar levels of 14915 protein (Fig. 3, bottom panel), indicating that the low amount and absence of the protein in the supernatants of the WT strain and the *lspF* mutant were not due to an unexpected lack of gene expression. Thus, 14915 is a secreted protein and substrate of the *L. pneumophila* T2SS. We designated the corresponding gene/protein as *irsA*/IrsA.

### IrsA promotes the growth of *L. pneumophila* in low-iron media

**Fig 4:**
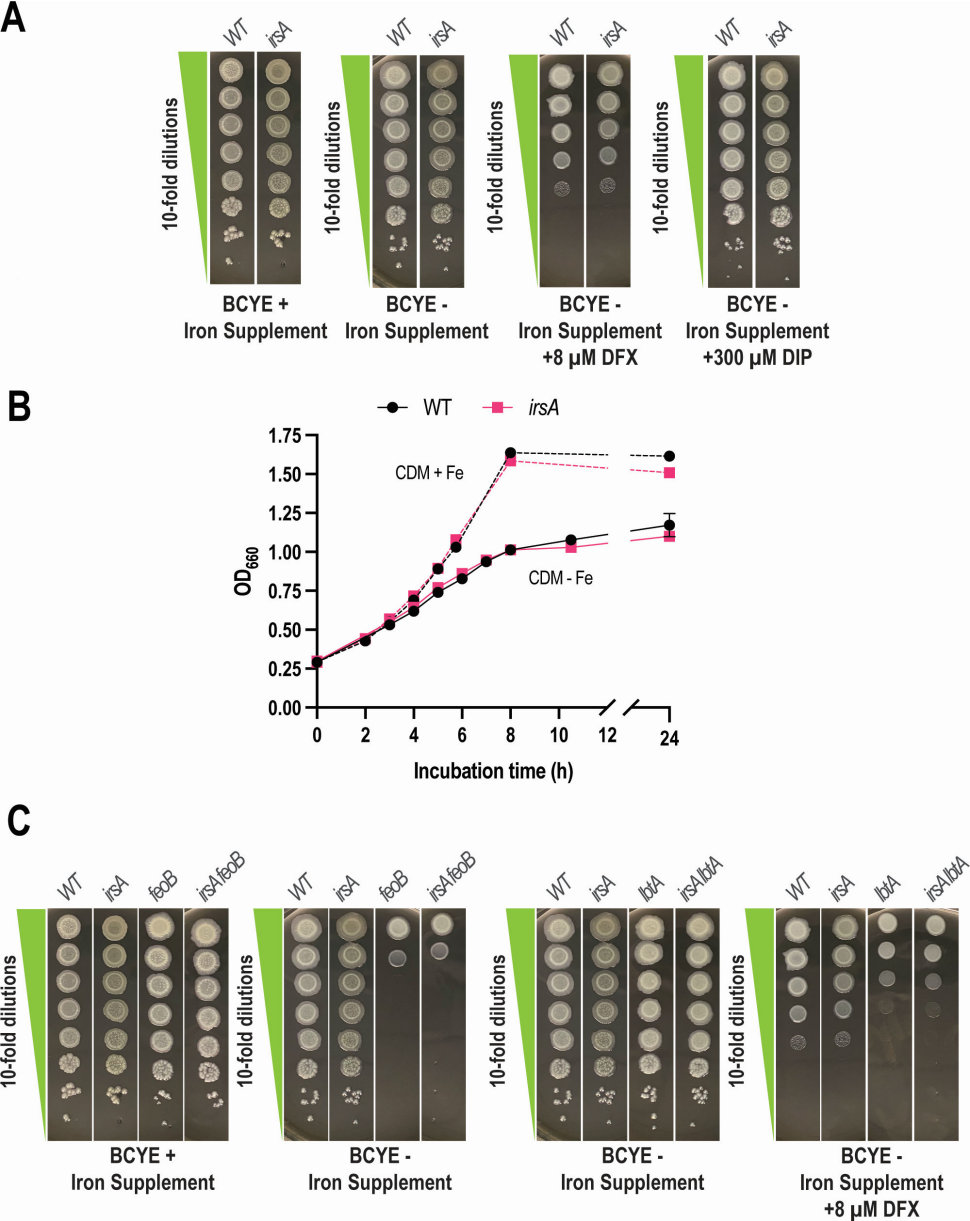
Effect of *irsA* mutations on the growth *L. pneumophila* strain 130b in bacteriological media containing decreasing amounts of available iron. (**A**) Following 3 days of growth on BCYE agar at 37°C, WT strain 130b (WT) and *irsA* mutant NU470 (*irsA*) were suspended in phosphate-buffered saline (PBS) to an OD_660_ = 0.3, and then 10 µL aliquots taken from a series of 10-fold dilutions were spotted onto BCYE agar that either contained its standard supplement of ferric pyrophosphate (+Fe supplement), lacked that iron supplement (−Fe supplement), lacked that supplement and instead had the iron chelator DFX added (−Fe supp + DFX), or lacked that supplement and instead had iron chelator DIP added (−Fe supp + DIP). Following incubation for 5 days at 37°C, images were taken of the areas of bacterial growth. (**B**) Following pre-culture in non-iron-supplemented BYE broth, WT 130b (circles) and *irsA* mutant NU470 (squares) were inoculated into either CDM that did not have any added iron (CDM − Fe, solid lines) or CDM that had 5 µM ferric pyrophosphate added (CDM + Fe, dashed lines) and incubated at 37°C with shaking. At the indicated times, bacterial growth was monitored spectrophotometrically. The data presented are the means and standard deviations from six technical replicates. (**C**) Following 3 days of growth on BCYE agar at 37°C, WT strain 130b (WT), *irsA* mutant NU470 (*irsA*), *feoB* mutant NU458 (*feoB*), *irsA feoB* mutant NU474 (*irsA feoB*), *lbtA* mutant NU302 (*lbtA*), and *irsA lbtA* mutant NU472 (*irsA lbtA*) were resuspended in PBS to an OD_660_ = 0.3, and then 10 µL aliquots taken from a series of dilutions were spotted onto either BCYE agar that had or lacked its added iron supplement (for comparing the *feoB* mutant to the *irsA feoB* mutant in the two left-most panels) or BCYE agar that lacked the iron supplement and had or lacked added DFX (for comparing the *lbtA* mutant to the *irsA lbtA* mutant in the two right-most panels). The images in panels (**A**) and (**C**) derive from the same representative experiment. However, the comparisons of WT and the *irsA* mutant to the other mutants were placed in the separate (**C**) to make the comparisons between the many strains easier to discern. Thus, the images for WT and the *irsA* mutant on BCYE + Fe supp., BCYE – Fe supp., and BCYE – Fe supp. + DFX are duplicated in (**A**) and in the different parts of (**C**). For (A–C), the presented data are representative of the results from >3 independent experiments.

Since *14915*/*irsA* was uncovered because of its hyperexpression in low-iron conditions, we posited that its protein product promotes *L. pneumophila* iron acquisition. To begin to test this hypothesis, we constructed a mutant of strain 130b that specifically lacks *irsA* and then tested the new mutant for growth in low-iron media. The *irsA* mutant grew similarly to parental WT when it was plated onto buffered charcoal yeast extract (BCYE) agar, the nutrient-rich, solid medium that is traditionally used to grow *L. pneumophila* (71). However, the mutant also showed a normal efficiency of plating when grown on BCYE agar that either lacked its usual iron supplement (i.e., ferric pyrophosphate) or was further depleted of available iron by the inclusion of the ferric iron chelator DFX or the ferrous iron chelator di-pyridyl (DIP) (Fig. 4A). The *irsA* mutant of strain 130b also grew comparably to its parental strain when they were inoculated into CDM – Fe broth (Fig. 4B). These results indicated that IrsA is not required for the growth of strain 130b in commonly used low-iron conditions. We considered the possibility that IrsA might be functionally redundant with ferri-rhizoferrin and FeoB-mediated ferrous iron transport, *L. pneumophila*’s main modes of iron assimilation during growth on bacteriological media (14). To test this possibility, we constructed and tested a mutant of strain 130b lacking both *irsA* and *lbtA* as well as a 130b mutant lacking both *irsA* and *feoB*. However, when cultured on low-iron BCYE agar (Fig. 4C) or in CDM − Fe broth (Fig. S4), the *irsA lbtA* mutant grew comparably to the *lbtA* mutant and the *irsA feoB* mutant grew similarly to the *feoB* mutant, indicating that IrsA is not functionally redundant with ferri-rhizoferrin or FeoB under these standard growth conditions.

**Fig 5:**
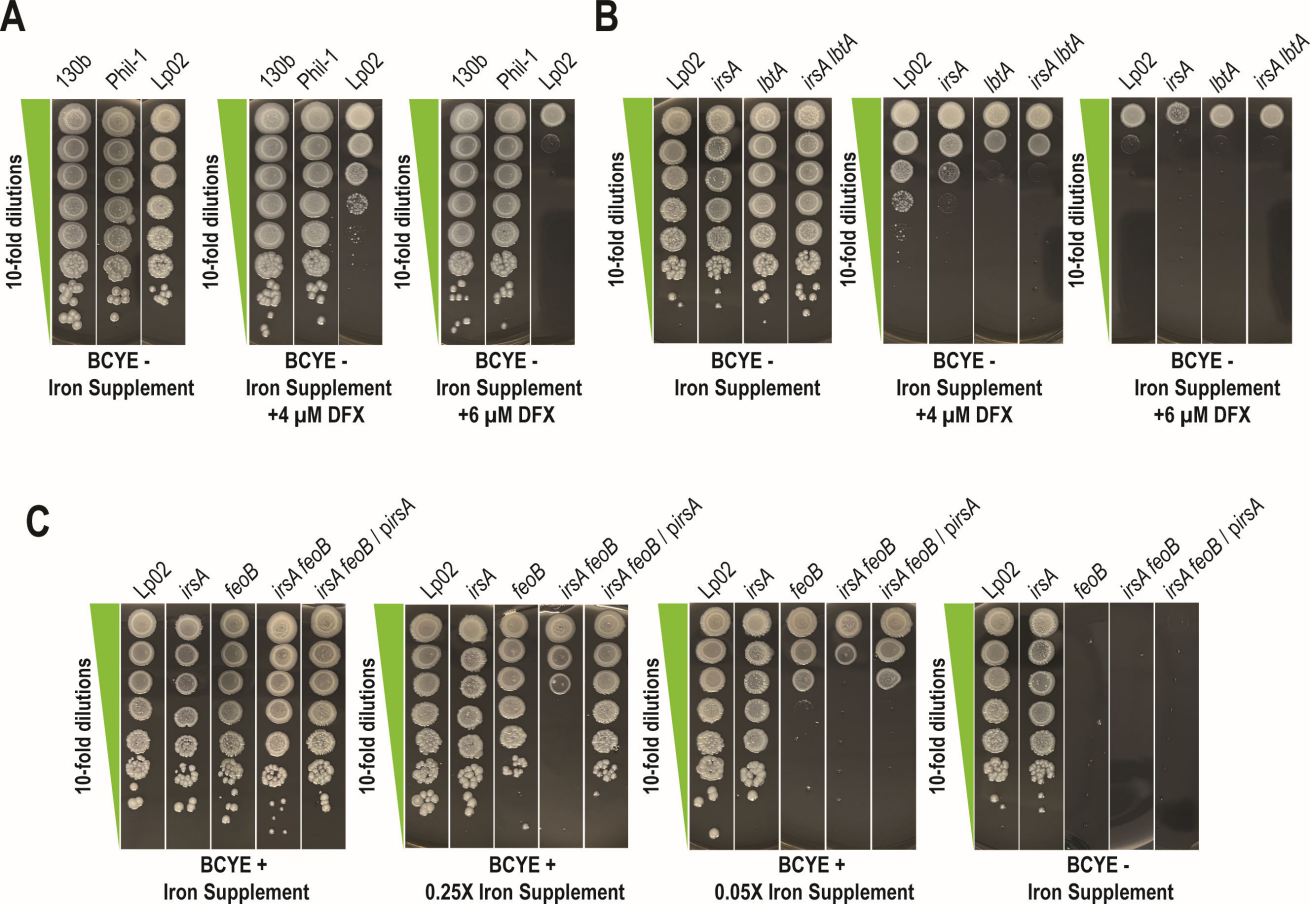
Relative growth of *L. pneumophila* strain Lp02 on low-iron media and the effect of *irsA* mutations on the growth of Lp02 on low-iron media. (**A**) Following growth on thymidine-supplemented BCYE agar, WT strain 130b (130b), WT strain Philadelphia-1 (Phil-1), and the Phil-1 derivative Lp02 (Lp02) were suspended in PBS to an OD_660_ = 0.3, and then 10 µL aliquots taken from a series of 10-fold dilutions were spotted onto thymidine-supplemented BCYE agar that either lacked the standard ferric pyrophosphate supplement (BCYE − iron supplement) or lacked the supplement and instead had increasing concentrations of the ferric iron chelator DFX added (BCYE − iron supplement + DFX). Following incubation for 5 days at 37°C, images were taken of the areas of bacterial growth. (**B**) Following growth on thymidine-supplemented BCYE agar, strain Lp02 (Lp02) and its derivatives that additionally lacked either *irsA* (i.e., strain NU476) (*irsA*), *lbtA* (NU480) (*lbtA*), or *irsA* and *lbtA* (NU484) (*irsA lbtA*) were spotted onto thymidine-supplemented BCYE agar that had decreasing amounts of available iron as indicated in (**A**) and their subsequent growth monitored as above. (**C**) Following growth on thymidine-supplemented BCYE agar, strain Lp02, its derivatives that also lacked either *irsA*, *feoB* (i.e., NU478) (*feoB*), or *feoB* and *irsA* (NU482) (*irsA feoB*), and NU482 containing a plasmid-carried *irsA* gene (*irsA feoB* + p*irsA*) were spotted (as above) onto thymidine-supplemented BCYE agar that either had the standard ferric pyrophosphate supplement (BCYE + iron supplement) or had only 25%, 5%, or 0% of the ferric pyrophosphate added along with 0.5 mM IPTG. The images in panels (A–C) derive from the same representative experiment. However, the comparisons of Lp02 to its various mutant derivatives were placed in the (**B**) and (**C**) to make the comparisons between the many strains easier to discern. Thus, the image for Lp02 on BCYE – Fe supp. + 6 µM DFX is duplicated in (**A**) and (**B**), and the images for Lp02 and its *irsA* mutant on BCYE – Fe supp. are duplicated in (**B**) and (**C**). For (A–C), the presented data are representative of the results from >3 independent experiments.

A commonly used strain in the *L. pneumophila* field is strain Lp02, which is a laboratory-derived variant of the clinical isolate Philadelphia-1 (72–80). Prompted by our long-standing interest in assessing the iron requirements and modes of iron acquisition of different strains of *L. pneumophila* (12, 14, 15, 17, 18, 24, 26, 29, 31, 32, 35, 38, 40, 41, 44), we discovered that strain Lp02 exhibits an ~100- to 10,000-fold reduced efficiency of plating on low-iron BCYE agar media relative to its parent Philadelphia-1 and WT strain 130b (Fig. 5A). Incidentally, impaired growth on low-iron media was not observed when we tested strain JR-32, which is another commonly used derivative of Philadelphia-1 (81) (Fig. S5). We suspected that Lp02’s impaired growth on low-iron media was due, at least partly, to the ~45 kb deletion (encompassing the *lvh* T4SS, among other loci) and/or one or more of the point mutations or small deletions (affecting *rpsL, ndh, leuS, nuoG,* and *thyA*) that exist in the Lp02 genome (81–83). Since the reported mutations in Lp02 do not encompass rhizoferrin-associated genes (*lbtABC, lbtU*), the ferrous transport operon (*feoABC*), the other genes that were most highly induced by iron deprivation (*frgA*, *mavN/iroT*), or other genes that we found promote *L. pneumophila* growth on low-iron media (e.g., *ccmC, iraB, lbtP, lly, tatB*) (17, 29, 36, 40, 43), we reasoned that Lp02 lacks an uncharacterized factor(s) that promotes iron assimilation, and therefore, the strain might prove useful for uncovering a role for *irsA*. Thus, we generated an *irsA* mutant of strain Lp02 and tested it for growth on agar media with decreasing amounts of available iron. However, the new mutant did not display impaired growth on BCYE agar lacking the usual iron supplement, and only showed a relatively modest defect on some non-iron-supplemented media that had added iron chelator (Fig. 5B). Adopting the logic that we had applied for studying strain 130b, we made and tested an Lp02 mutant lacking both *irsA* and *lbtA*. This double mutant was no more impaired for growth on the low-iron BCYE agar than was the Lp02 mutant lacking only *lbtA* (Fig. 5B). However, a newly made Lp02 mutant lacking both *irsA* and *feoB* was significantly more impaired for growth on low-iron BCYE agar than was an Lp02 mutant lacking only *feoB*, e.g., the double mutant had a 100- to 1,000-fold reduced efficiency of plating on BCYE agar having only one-quarter of its usual iron supplement (Fig. 5C). Moreover, the growth defect of the Lp02 *irsA feoB* mutant was reversed when we reintroduced an intact copy of *irsA* (Fig. 5C). Thus, IrsA promotes *L. pneumophila* growth in low-iron media, at least under some circumstances.

### IrsA impedes or down-regulates *L. pneumophila* biofilm formation

**Fig 6:**
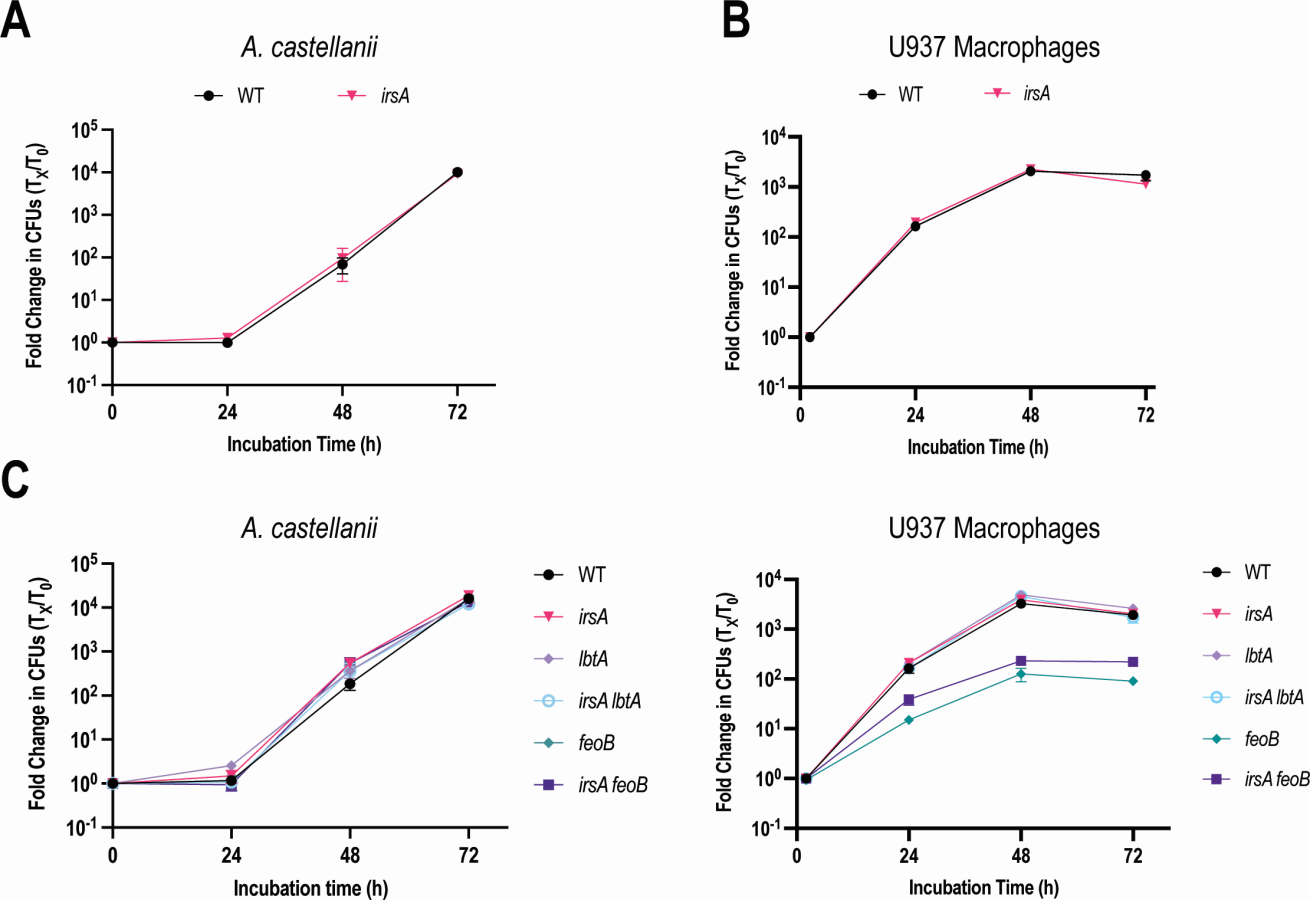
Effect of *irsA* mutations on infection of amoebae and human macrophages by *L. pneumophila* strain 130b. (**A**) *A. castellanii* were infected with either wild-type strain 130b (WT) or the *irsA* mutant NU470 (*irsA*), and then at 0, 24, 48, and 72 h post-inoculation, net bacterial growth (number of CFU at the denoted time/number of CFU at *t* = 0) was determined by plating aliquots of the supernatant on BCYE agar. Since *L. pneumophila* does not grow in the assay medium, any increases in CFU are the result of growth in the acanthamoebae. (**B**) Monolayers of differentiated U937 cells were inoculated with WT or the *irsA* mutant NU470. After a 2-h incubation to allow for bacterial entry, the infected wells were washed to remove remaining extracellular bacteria, and then at 0, 24, 48, and 72 h post-inoculation, the macrophages were lysed, bacterial numbers in the infected wells determined by plating, and net bacterial growth obtained as above. Since *L. pneumophila* does not grow in the assay medium, any increases in CFU are the result of growth within the macrophages. (**C**) WT, *irsA* mutant NU470, *lbtA* mutant NU302 (*lbtA*), *irsA lbtA* mutant NU472 (*irsA lbtA*), *feoB* mutant NU458 (*feoB*), and *irsA feoB* mutant NU474 (*irsA feoB*) were used to infect *A. castellanii* (left) or differentiated U937 cells (right), and net bacterial growth determined as above. In (A–C), the values presented are the means and standard deviations obtained from three technical replicates and are representative of the results obtained from three independent experiments.

**Fig 7:**
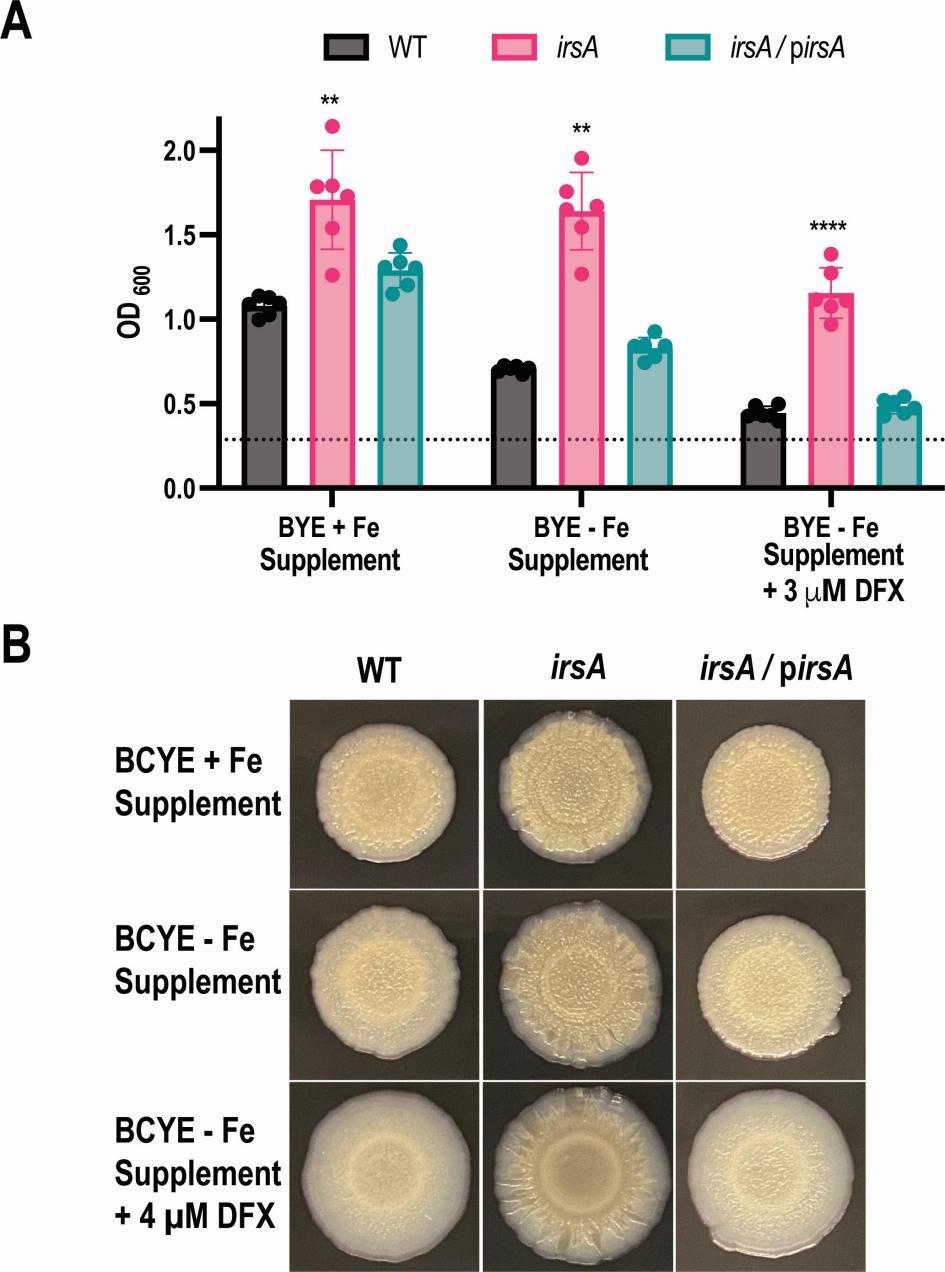
Effect of IrsA on biofilm formation by *L. pneumophila* strain 130b. (**A**) Following 3 days of growth on BCYE agar without iron supplement at 37°C, WT strain 130b (WT), *irsA* mutant NU470 (*irsA*), and NU470 containing a plasmid-carried *irsA* gene (*irsA*/p*irsA*) were resuspended to an OD_660_ of ~0.2 in either BYE broth containing its customary ferric pyrophosphate supplement (BYE + Fe supplement), BYE broth lacking that iron supplement (BYE – Fe supplement), or BYE broth that lacked the iron supplement but had added 3 µM DFX (BYE – Fe supp + 3 µM DFX), and then the suspensions were added into the wells of a 96-well, plastic microtiter plate. After 2 days at 30°C, the amount of biofilm formed was determined by staining with crystal violet as read at 600 nm. The reading obtained from control wells containing only medium is indicated by the horizontal dashed line. Data presented are the means and standard deviations from six technical replicates and are representative of three independent experiments. Asterisks indicate differences in the levels of biofilm formation between the mutant and the other two strains: **, *P* < 0.01; ****, *P* < 0.0001. (**B**) Bacterial strains, as indicated in (**A**), were spotted onto the surface of BCYE agar either containing either its customary iron supplement (+ Fe supplement), lacking that iron supplement (– Fe supplement), or lacking the iron supplement but instead containing 4 µM (– Fe supplement + DFX). Following incubation for 8 days at 37°C, images were taken of the areas of bacterial growth. The presented data are representative of the results from three independent experiments.

**Fig 8:**
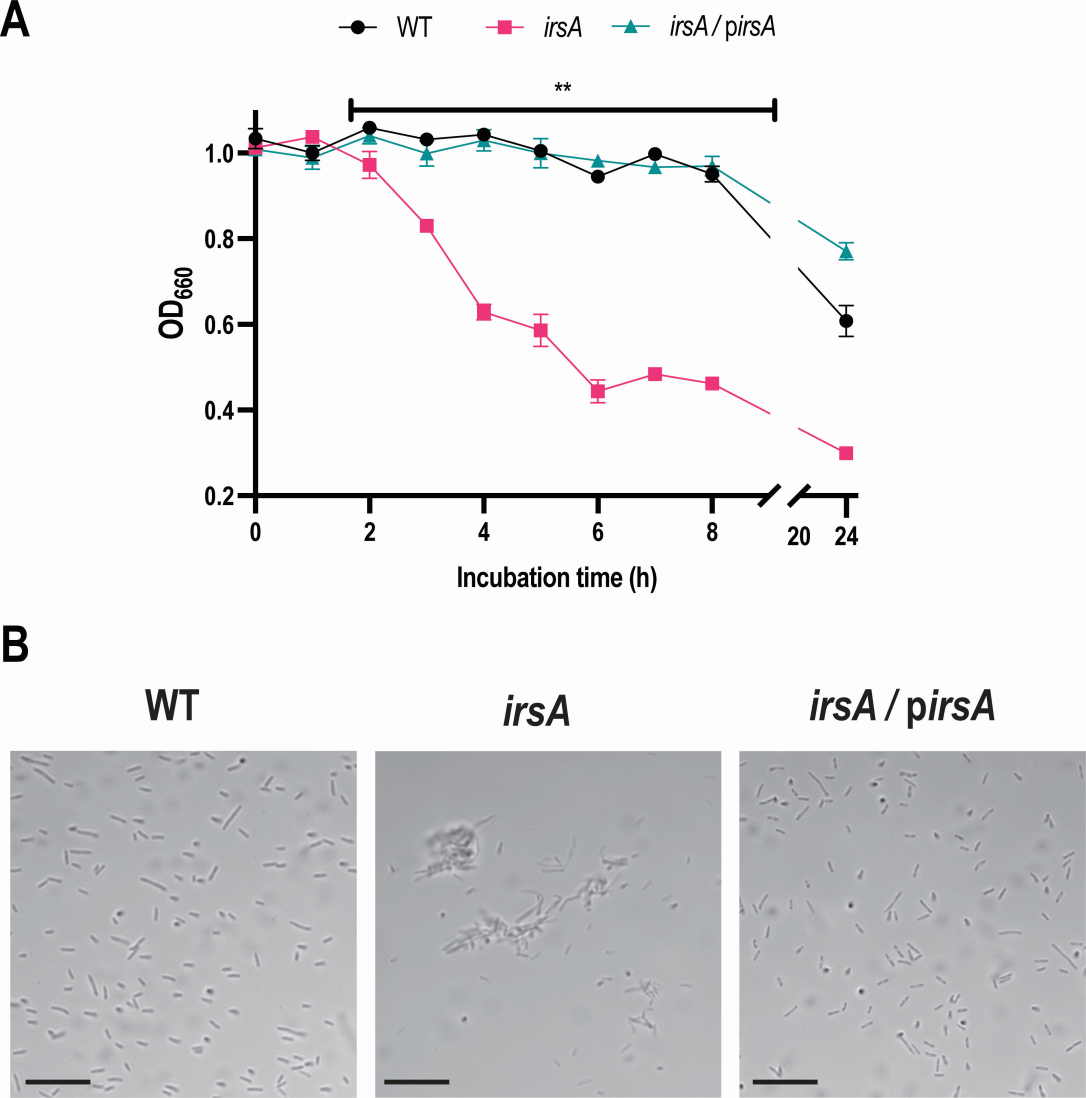
Effect of IrsA on auto-aggregation by *L. pneumophila* strain 130b. (**A**) Following 3 days of growth on BCYE agar at 37°C, WT strain 130b (WT), *irsA* mutant NU470 (*irsA*), and NU470 containing a plasmid-carried *irsA* gene (*irsA*/p*irsA*) were suspended in 10% BYE broth to an OD_660_ of ~1.0. Five-milliliter aliquots were added to glass tubes, and bacterial sedimentation at 30°C was assessed by measuring drops in the OD_660_ of the suspensions. The data presented are the means and standard deviations from three technical replicates and are representative of three independent experiments. Asterisks indicate that the mutant behaved differently from the other two strains at all time points after 2 h: **, *P* < 0.01. (**B**) Following static incubation, as indicated in (**A**), strain aggregation was assessed at *t* = 4 h by microscopically examining an aliquot taken from the mid-point in the tube. The scale bar corresponds to 10 µm.

To begin to gauge the impact of IrsA on the environmental persistence and pathogenesis of WT strains of *L. pneumophila*, we examined the mutants of strain 130b lacking *irsA* for their capacity to infect amoebae and macrophages and to form biofilms. Neither the *irsA* mutant nor the *irsA lbtA* mutant was defective for infection of *Acanthamoeba castellanii* or human U937 cells (Fig. 6), indicating that IrsA is not required for optimal intracellular infection by *L. pneumophila*, even in the absence of rhizoferrin synthesis. The *irsA feoB* mutant was no more impaired for infection than was the *feoB* mutant (Fig. 6), indicating that IrsA is also not required for intracellular infection by strain 130b in the absence of FeoB-mediated ferrous transport. However, the *irsA* mutant exhibited a higher level of biofilm formation on plastic surfaces than its parent did, and this was observed whether the legionellae were incubated in BYE broth (Fig. 7A) or in CDM broth (Fig. S6). Moreover, the hyper-biofilm phenotype of the *irsA* mutant was seen across a range of available iron concentrations (Fig. 7A; Fig. S6). The heightened biofilm levels shown by the mutant were reversed upon reintroduction of an intact *irsA* gene (Fig. 7A), confirming that IrsA impedes or down-regulates biofilm formation by *L. pneumophila*. Since the *irsA* mutant of strain 130b grew typically in the media that were used in these assays (above), the greater amount of biofilm observed here was not simply due to greater numbers of mutant bacteria following introduction into the plastic wells. With *L. pneumophila* and others, biofilm-linked phenotypes can also be observed by spotting an aliquot of bacteria unto an agar surface and then observing the pattern of outgrowth over the surface (14, 84, 85). When we performed this assay, the *irsA* mutant, but not its complemented derivative, exhibited a central area of growth that conveyed more elaborate structures (Fig. 7B). The extent of the mutant’s biofilm-like pattern was more dramatic and most clearly discerned on those agar plates that contained lower amounts of available iron (Fig. 7B), a result that is compatible with the induction of *irsA* expression in low-iron (Fig. 1 and 2). Considering these findings, we next examined whether the *irsA* mutant also displays increased auto-aggregation. Like many other bacteria, *L. pneumophila* gradually forms auto-aggregates when it is suspended in non-agitated liquid in a glass tube, with the bacterial cells eventually settling to the bottom of the tube, thereby reducing the suspension’s optical density (OD) (86, 87). Upon 1–8 h of incubation in 10% BYE broth, a suspension of WT 130b did not show a decrease in OD; however, after 24 h of incubation, evidence of aggregation was seen, as expected (Fig. 8A). In marked contrast, the *irsA* mutant, but not its complemented derivative, appeared to aggregate as early as 3 h after inoculation, and the OD of the mutant’s suspension continued to rapidly decrease over the next 5 h (Fig. 8A). Moreover, the mutant phenotype persisted out to the 24-h endpoint of the experiment (Fig. 8A). The mutant’s apparent hyper-aggregation was also evident by visual detection of a sediment in the bottom of the tubes (data not shown). When the tubes containing the bacteria were vortexed, the OD of the *irsA* mutant’s suspension matched that of WT, further indicating that the mutant’s phenotype was not simply due to its increased lysis. Finally, the hyper-aggregated nature of the *irsA* mutant was evident by microscopic examination (Fig. 8B). Taken together, these data indicated that IrsA impedes or down-regulates biofilm formation and auto-aggregation by *L. pneumophila*. We hypothesize that the hyper-aggregative nature of the *irsA* mutant explains, at least in part, the hyper-biofilm phenotype of the mutant.

## DISCUSSION

First and foremost, the RNA-Seq analysis that began the current study allowed us to uncover IrsA, which, based on subsequent experimentation, is an iron-regulated/repressed, secreted protein that appears to have a novel role in *L. pneumophila* growth and biofilm formation. As mentioned earlier, *irsA* (expression) had not been detected in the one prior transcriptomic analysis of *L. pneumophila* grown in an iron-deplete medium (vs iron-replete medium) (18). We suspect that this most likely occurred because that past study examined *L. pneumophila* grown in a yeast extract broth containing added iron chelator but was otherwise rich with nutrients, whereas our study examined *L. pneumophila* grown in a chemically defined broth that not only lacked added iron but consisted of only free amino acids and other trace metals. Thus, we hypothesize that, during planktonic growth, *irsA* expression mostly occurs when both iron levels and other (complex) nutrients levels are low. However, it is also conceivable that *irsA* was previously overlooked because the past study employed microarrays to detect transcripts rather than the RNA-Seq approach used here. Looking beyond *irsA*, our RNA analysis further documented, by another method, the often-large degree to which iron levels modulate the expression of the known rhizoferrin-related genes, the *feoB* operon, *frgA*, *mavN/iroT*, and the *sufA* operon linked to iron-sulfur cluster proteins (12, 18, 19, 26, 28, 29, 32). The present study also identified a variety of other genes that are induced during *L. pneumophila* growth in low-iron conditions (Fig. 1B). Some of these, including *nrdB*/*rir1* and *nrdA*/*rir2* that encode a ribonucleotide-diphosphate reductase, had been revealed in the prior microarray study (18). More interestingly, however, the current RNA-Seq analysis uncovered a variety of other genes (besides *irsA*) whose induction by low iron had not been observed before. Notable among these was a gene annotated as *cyaA*, which maps directly downstream of *lbtC* and is predicted to encode an inner membrane spanning, class III adenylate cyclase that has a histidine kinase sensor domain (88, 89). Given its 2.9-fold induction by low-iron conditions (Fig. 1B), proximity to the *lbtABC* operon, and the fact that some adenylate cyclases are linked to the regulation of siderophores and/or other forms of bacterial iron uptake (90, 91), we hypothesize that this gene (and by extension, cAMP levels) influences rhizoferrin and/or another mode(s) of *L. pneumophila* iron acquisition. Also notable among the genes that were newly identified as being induced (two- to fivefold) by low-iron conditions (Fig. 1B) are (i) a set of genes related to biotin synthesis, suggesting that another form of iron-sulfur cluster biogenesis might be occurring in *L. pneumophila* (92), (ii) *hspC2*, which we recently determined promotes both thermal tolerance and intracellular infection (93), and (iii) *chiA*, which we previously showed encodes a T2SS-dependent chitinase/mucinase that can promote lung infection (60, 94). Finally, and also significant for a further understanding of infection by *L. pneumophila*, our transcriptomic analysis revealed 18 T4SS effector genes (i.e., *ceg18, ravN, lem27, ceg7, ravI, lpg2395, ceg29, lgt3/legC5, lpg1106, ceg23, sidD, legC3/ppeA, lem13/ppeB, ceg14/sidL, ceg22, vipE, ravK, legA7/ankZ*) (63, 95–113) that are induced ~2- to 4-fold by low-iron conditions (Fig. 1B). Thus, the impact of low-iron growth conditions on the expression of T4SS effectors extends well beyond the previously described induction of MavN/IroT (18–20). Yet, many of these genes, unlike *mavN/iroT*, do not have a Fur box in their promoter regions, implying that their regulation by iron is indirect and (also) involves another transcriptional regulator(s) and environmental signal(s). Thus, in addition to launching further studies of IrsA, the results obtained here provide a springboard for multiple, other lines of inquiry.

Given that bacteria generally have many modes of iron (metal) assimilation and that the importance of a given assimilation pathway is often only evident when other pathways are disabled by mutation, it is not illogical that the role of IrsA in promoting *L. pneumophila* growth in low-iron conditions was discerned through mutation of Lp02, a lab-adapted strain that is known to harbor a variety of mutations in its genome (81, 83) and, as we documented here, has difficulties growing on low-iron media. Since the role of *irsA* in the Lp02 background was especially revealed by finding a greater growth defect on low-iron BCYE agar for an *irsA feoB* mutant (vs an *feoB* mutant), we posit IrsA has a role that is functionally complementary to FeoB/Fe^2+^ uptake and thus may entail the assimilation of Fe^3+^ or heme/hemin-containing compounds in the medium’s yeast extract. Because an *irsA lbtA* mutant of Lp02 was not different from an *lbtA* (rhizoferrin synthesis) mutant of Lp02, we infer that any possible role for IrsA in ferri-siderophore acquisition does not involve rhizoferrin but rather the activity of a yet-to-be-defined siderophore. Although a role for IrsA in iron acquisition would appear to be the simplest explanation for both the induction of *irsA* expression when *L. pneumophila* is grown in iron-deplete media and the growth defect of the Lp02 *irsA feoB* mutant, it is also conceivable that IrsA is involved in the acquisition of other metals that might substitute for iron as a cofactor when the available iron levels are too low (114). Finally, it is possible that IrsA is not involved in the acquisition of iron or other metal(s) but in some novel way helps *L. pneumophila* adapt its physiology to low-iron conditions. Given that there are no characterized homologs of IrsA in other bacteria, future studies aimed at deciphering the action of IrsA should prove highly innovative. It will be informative to also discern which of the many gene losses in strain Lp02 are responsible for that strain’s poorer growth on low-iron media, as another possible means toward unearthing new factors involved in iron assimilation.

Even though this study achieved its original goal of identifying a new factor involved in *L. pneumophila*’s growth in low-iron conditions, the most dramatic effect of IrsA was observed in the context of biofilm formation. Indeed, mutation of *irsA* alone resulted in a marked increase in biofilm formation, and moreover, this effect was seen in an otherwise virulent, WT strain (130b) background. Thus, IrsA appears to either directly impede or indirectly down-regulate biofilm formation. Based on our additional phenotyping of the *irsA* mutant, we suspect that this effect is due at least in part to IrsA inhibiting *L. pneumophila* auto-aggregation. These data suggest that IrsA may, in some situations, modify the surface traits of *L. pneumophila*. Curiously, the increased biofilm formation and hyper-aggregation phenotype of the *irsA* mutant (but not its complemented derivative) was evident across a range of iron levels, implying that the iron/Fur-mediated repression of *irsA* is changed or not entirely operative in biofilms. We have not observed this sort of dis-linkage before, i.e., *feoB* and *lbtA feoB* mutants of strain 130b that were impaired for iron acquisition and growth in low-iron bacteriological media were also impaired for biofilm formation on plastic and agar surfaces (14). Thus, in the context of biofilms, the expression of *irsA* may be (more) subject to other environmental signals or regulators, which could include other metals and nutrients, c-di-GMP, quorum-sensing, and oxidative stress, among others (3, 115–117). Hence, it will be exciting for future investigations to also explore IrsA expression within biofilms and investigate how this secreted protein modulates cell-cell interactions and biofilm formation.

The identification of IrsA as a substrate of the *L. pneumophila* T2SS represents a notable new connection between iron-related functions and biofilms and the bacterial T2SS. Indeed, prior to our study, the number of such linkages has been relatively few (118). In the case of iron-related functions and T2SS, there are the instances of degradation of host transferrin by the *L. pneumophila* exoprotein ProA, the degradation of hemoglobin by VvpE secreted by *Vibrio vulnificus*, the impact of a secreted lipase on pyoverdine regulation in *Pseudomonas aeruginosa*, a possible connection to siderophore receptors in *Pseudoalteromonas tunicata*, the reduction of Fe^3+^ by *Shewanella oneidensis*, and the secretion of iron-binding IbpS which provides protection to *Dickeya dadantii* against reactive oxygen species (42, 118–123). Regarding past connections between biofilms and T2SS, there are examples of the secretion system promoting biofilm formation by *Acinetobacter baumannii*, enteropathogenic *Escherichia coli, L. pneumophila, Marinobacter hydrocarbonoclasticus, P. aeruginosa,* and *Vibrio cholerae* (118, 124–128). For *L. pneumophila*, the Lcl protein has been identified as facilitating biofilm formation (129, 130). Thus, the relationship between the *L. pneumophila* T2SS and biofilm formation is a complex one, with some exoproteins (e.g., Lcl) promoting and others (e.g., IrsA) impeding biofilm formation. Undoubtedly, the T2SS provides *L. pneumophila* with the ability to adapt to diverse growth conditions that exist in its environment. The documentation of IrsA as a substrate of the *L. pneumophila* T2SS also brings the number of characterized *L. pneumophila* T2SS-dependent exoproteins to 26, with still dozens more being predicted by *in silico* analysis and proteomic assessments of WT culture supernatants (8, 60–63). That IrsA was encoded within the genomes of ~30% of other *Legionella* species is compatible with what we have seen for known T2SS substrates, whose prevalences across the genus range from 11% to 100% (8). However, the fact that many of these other species are known to be pathogenic for humans (Fig. 2C) reinforces the argument for further investigation into IrsA function. Although they are presently few in number, the IrsA-related proteins encoded by non-*Legionella* bacteria are also similarly noteworthy, e.g., these proteins might impact the pathogenesis of *P. salmonis*, a major pathogen of fish, and/or *C. burnetii*, the zoonotic agent of human Q fever (131, 132). Previously, we observed that some of the T2SS substrates of *L. pneumophila* have eukaryotic-like domains, with the related proteins in eukaryotes occurring in fungi, algae, amoebae, and cnidarians (8). Thus, the detection of IrsA-related proteins in rotifers and dinoflagellates provides a new example of the intriguing relationship between the secreted proteins of *L. pneumophila* and proteins in environmental eukaryotes.

## MATERIALS AND METHODS

### *L. pneumophila* strains, bacteriological media, and extracellular growth assays

*L. pneumophila* WT strain 130b (American Type Culture Collection [ATCC] strain BAA-74; also known as AA100 or Wadsworth) was previously described as were its *lbtA* mutant NU302, *feoB* mutant NU458, *proA* mutant AA200, and *lspF* mutant NU275 (14, 26, 70, 133). Other previously described strains that were used in this study were *L. pneumophila* WT strain Philadelphia-1 (ATCC 33152) (134), strain Lp02, a laboratory-generated variant of Philadelphia-1 (9, 72), and strain JR-32, another derivative of Philadelphia-1 (81). These various strains and all newly made *L. pneumophila* mutants (below) were routinely grown at 37°C on BCYE agar or in BYE broth (71). In order to assess the response of WT and mutant *L. pneumophila* to variations in available iron, bacteria were inoculated into CDM broth (22–25) containing differing amounts of added iron and then either the extent of growth was determined by changes in the OD_660_ of the cultures, as previously done (14), or the changes in gene expression were examined as experimentally detailed below. As before (14, 28, 29), bacteria were also examined for their efficiency of plating on BCYE agar that either contained its standard added iron supplement (i.e., 330 µM ferric pyrophosphate), contained 25% or 5% of that iron supplement, entirely lacked that supplement, or lacked that supplement and instead had increasing amounts of the iron chelators DFX or DIP added. Unless otherwise noted, chemicals were from Sigma-Aldrich.

### Measurement of iron levels

Quantification of iron in CDM broth containing no added iron (CDM – Fe) was accomplished using ICP-MS, as we previously did for non-iron-supplemented BYE broth (14, 135). On three occasions, 1.0 mL of CDM that had no added iron (CDM – Fe) was treated with concentrated trace-grade nitric acid at 65°C to allow for complete digestion. Ultra-pure H_2_O was then added to produce a solution of 5% nitric acid in a total volume of 10 mL. A 100 ng/g mixed element standard including Fe was made in 5% nitric acid (vol/vol) to a total volume of 50 mL. ICP-MS was done on a computer-controlled (QTEGRA software) Thermo iCapQ ICP-MS operating in KED mode and equipped with an ESI SC-2DX PrepFAST autosampler. Internal standard was added in-line using the PrepFAST system. Online dilution was carried out by the PrepFAST system and used to make a calibration curve consisting of 100, 50, 25, 10, 2, and 1 ppb Fe. Each sample was acquired using one survey run (10 sweeps) and three main (peak jumping) runs (40 sweeps). Isotopes selected for analysis were ^56,57^Fe, and, as internal standards for data interpolation and machine stability, ^45^Sc, ^89^Y, and ^115^In. The Fe levels obtained for the three replicates were 0.1573 µM, 0.1315 µM, and 0.1319 µM, giving an average value of 0.1402 µM.

### Complete genome sequencing of *L. pneumophila* strain 130b

We determined the complete and fully assembled genome sequence of strain 130b, analogously to what we recently did for the genome of *Legionella cardiaca* strain H63T (136). Following the growth of strain 130b on standard BCYE agar, genomic DNA (gDNA) was isolated using the Promega Maxwell 16 system. The gDNA was then sequenced using Illumina and PacBio platforms. For the Illumina platform, short-read libraries were made with a Roche KAPA HyperPrep kit and sequenced using 150 bp paired-end reads on the NovaSeq 6000 system. For the PacBio platform, the gDNA was fragmented to an average size of approx. 11 kb with Covaris g-TUBE. After the DNA was cleaned with SPRIselect beads, library construction proceeded using the SMRTbell Express Template Prep Kit 2.0 (PacBio), which comprises ss-DNA overhang removal, damage repair, end repair/A-tailing, and barcoded overhang adaptor ligation. The library was pooled with other libraries on an equimolar basis and then size selected on a BluePippin instrument with an 8 kb cutoff value. The pool was then purified with SPRIselect beads, quantified with a Qubit 4.0 fluorometer, and evaluated with an Agilent Fragment Analyzer. The final library pool was sequenced with PacBio Sequel II v.3.0 chemistry and a single-molecule real-time (SMRT) Cell 8M on a Sequel I instrument at an on-plate concentration of 85 pM. Illumina reads were quality filtered using a combination of Illumina RTA v.1.8.70.0 and Trimmomatic v.0.38.0 (137). PacBio reads were quality filtered using FastQC v.0.72 (138). For Illumina sequencing, 62,679,165 reads were generated with ~151× coverage, and for PacBio sequencing, there were 134,282 reads (N_50_, 13,379 bp). The resulting raw sequences were handled using PacBio SMRTLink v.6.0, including demultiplexing by Lima v.1.11.0 (139). Genome assembly was done using the PacBio HGAP4 assembler, which contains overlap determination, followed by consensus polishing by Pilon 1.24 (140) using Illumina 150 bp paired-end reads made from the same gDNA. Rotation of the chromosome was done using the IGS automated prokaryotic annotation pipeline (141). The assembly yielded a single closed, circular chromosome. Gene annotation was done using the NCBI Prokaryotic Genome Annotation Pipeline v.6.4 (142–144). The *L. pneumophila* strain 130b genome assembly contains a single 3,538,602 bp contig (~37.92× coverage) with a G + C content of 38.5% and is predicted to encode 3,193 proteins.

### qRT-PCR and RNA-Seq analyses

*L. pneumophila* WT strain 130b was grown at 37°C in CDM with 5 µM ferric pyrophosphate added (CDM + Fe) and CDM without any added iron (CDM – Fe) to either mid-log (OD_660_ = 0.7), late-log (OD_660_ = 1.0), or early-stationary (OD_660_ = 1.5), and then total RNA was isolated using the GeneJet RNA purification kit (Thermo Fisher) as previously done (93). qRT-PCR was performed as before (93). Primers used for the current qRT-PCR analysis were designed using the Primer-BLAST tool on the NCBI website (145) for the *lbtA* (AL31 and AL32), *lbtU* (AL33 and AL34), *feoB* (AL35 and AL36), *frgA* (AL37 and AL38), and *14915*/*irsA* (AL39 and AL40) genes, and are listed in Table S3. Prior to the RNA-Seq analysis, the quality and quantity of the RNA samples were verified using the Agilent Bio-analyzer 2100. RNA-Seq libraries were generated using the Universal Prokaryotic RNA-Seq Library Preparation Kit (NuGEN), strictly according to the manufacturer’s instructions. This process included rRNA depletion, purification and fragmentation of the remaining RNA, cDNA synthesis, 3’ end adenylation, Illumina adapter ligation, library PCR amplification, and validation. Sequencing of the libraries, including the production of single-end, 75 bp reads, was done using the Illumina HiSEQ4000.The quality of the DNA reads was evaluated using FastQC (138). Adapters were trimmed and reads of poor quality or aligning to rRNA sequences were filtered. The cleaned reads were aligned to the reference genome using STAR (146). Read counts for each gene were calculated using HTseq-count (147) in conjunction with a gene annotation file obtained from NCBI accession number GCA_041734345.1, i.e., the complete and assembled genome sequence of strain 130b (see above). Normalization and differential expression were determined using DESeq2 (148). The cutoff for determining significantly differentially expressed genes (i.e., from CMD + Fe cultures vs CDM – Fe cultures) was an FDR-adjusted *P*-value less than 0.05.

### Immunoblot analysis of *L. pneumophila* culture supernatants

To track the secretion of 14915/IrsA, a 6xHis tag was added to the C-terminus of the protein. As a first step in making the plasmid construct encoding this tagged protein, primers AL65 and AL66 were used to amplify, from 130b genomic DNA, a ~484 bp fragment containing the intact *14915* ORF with 3´−6xHIS. After the PCR product was digested with EcoRI and KpnI, the *14915–6xHis* containing fragment was cloned into pMMB2002, a plasmid which encodes chloramphenicol (Cm) resistance and is stably maintained by *L. pneumophila* (28). PCR using the vector-specific primer pair OR77 and OR78 confirmed that the newly made plasmid (i.e., p*14915-6xHis*) carried *14915–6xHis*. Plasmid p*14915-6xHis* was electroporated (149) into the WT strain 130b, its *proA* mutant AA200, and its *lspF* mutant NU275, and transformants containing p*14915-6xHis* were obtained by plating on BCYE agar containing Cm. Following overnight growth in standard BYE broth, the strains encoding 14915–6xHis were inoculated to an OD_660_≅ 0.3 in 70 mL of BYE broth (in a 250 mL flask) containing 0.1 mM IPTG (in order to induce expression of the tagged protein) and then incubated at 37°C with shaking for 1 to 3 h. Supernatants were collected by centrifugation of the cultures at 5,000 × *g* for 15 min at room temperature, followed by filtration through 0.22 µm syringe filters (EMD Millipore). The cell-free supernatants were concentrated 20-fold by isopropanol precipitation, as previously described (150), and suspended in Laemmli sample buffer. The cell pellets resulting from the centrifugation of the cultures were also recovered and suspended in Laemmli buffer. Samples of the concentrated supernatants and cell pellets (corresponding to equal amounts of culture per strain examined) were subjected to SDS-PAGE and then immunoblotted first with a 1:10,000 dilution of rabbit anti-6xHis antibody (Invitrogen cat # PA1-983B) in 1% skim milk-Tris-buffered saline (TBST). The secondary antibody used was IRDye 680 goat anti-rabbit IgG antibody (LI-COR Biosciences) at a 1:20,000 dilution in milk-TBST. Treated membranes were imaged using LI-COR Biosciences Odessey Fc Imaging System, as before (151).

### Mutant constructions

New mutants of strain 130b that contain a deletion in *irsA* were constructed by allelic exchange, analogous to the generation of various other mutants of *L. pneumophila* (14, 93, 152). To begin, a DNA fragment consisting of an FLP recombination target-flanked kanamycin (Kn)-resistance cassette bounded on the one side by the ~600 bp of sequences upstream of *irsA* ORF and flanked on the other side by the ~600 bp of sequences downstream of the *irsA* ORF was synthesized (Twist Bioscience). Five micrograms of the linear DNA containing the mutagenized *irsA* allele was used for natural transformation of either WT strain 130b or a previously made *lbtA* mutant of 130b as indicated below. Bacteria putatively containing an inactivated *irsA* gene were obtained by plating the transformation mixture onto BCYE agar containing Kn. Verification of mutagenized *irsA* within the Kn-resistant colonies was done by PCR using the primer pair AL41 and AL42. Finally, following transformation with pBSFLP, a *sacB*-containing counterselectable vector that also encodes the Flp recombinase and a gentamicin (Gm)-resistance gene, mutants harboring an unmarked deletion within *irsA* (and lacking pBSFLP) were recovered by plating the transformation mixture onto BCYE agar containing 5% sucrose and were scored for loss of resistance to both Kn and Gm (93). The mutants were verified by sequencing of the PCR amplicon obtained using primers AL43 and AL44. The new *irsA* mutants obtained were designated as NU470 and NU471. To construct mutants lacking both *irsA* and *lbtA*, we introduced the mutagenized *irsA* allele (above) into the previously made *lbtA* mutant NU302 (26). The genotype of the *irsA lbtA* mutants (NU472, NU473) was verified by PCR using primers AL41 and AL42. To construct mutants lacking both *irsA* and *feoB*, we used the allelic exchange to introduce a mutagenized *feoB* allele (14) into *irsA* mutant NU470. The genotype of the *irsA feoB* mutants (NU474, NU475) was verified by PCR using primers AL25 and AL26.

New mutants of strain Lp02 that contain deletions in *irsA*, *feoB*, and/or *lbtA* were also constructed by using a form of allelic exchange. To begin, mutagenized alleles of *irsA* (i.e., *lpg0266*), *feoB* (*lpg2657*) and *lbtA* (*lpg1325*) were generated using overlap extension PCR (OE-PCR) as previously done (14, 93, 152). Approx. 800 bp fragments of the 5´ and 3´ regions flanking the target ORFs were PCR amplified from Lp02 gDNA using Platinum SuperFi II DNA polymerase (Thermo Fisher Scientific) and primers AL45 and AL46 for 5´ *irsA*, AL47 and AL48 for 3´ *irsA*, AL53 and AL54 for 5´ *feoB*, AL55 and AL56 for 3´ *feoB,* AL59 and AL18 for 5´ *lbtA*, and AL60 and AL61 for 3´ *lbtA*. A Kn-resistance cassette flanked by Flp recombination target sites was PCR-amplified from pKD4 using primers AL49 and AL50 for use in the eventual mutation of *irsA*, AL57 and AL58 for eventual mutation of *feoB,* or AL23 and AL62 for eventual mutation of *lbtA*. We then performed two-step OE-PCR to combine the 5´ and 3´ regions of *irsA,* the 5´ and 3´ regions of *feoB,* and the 5´ and 3´ regions of *lbtA* with the respective Kn-resistance cassettes. PCR products corresponding to the correct target size were gel purified, and 1 µg of linear DNA containing the recombinant allele carrying the antibiotic cassette was used for natural transformation of either Lp02 or a mutant of Lp02 as indicated below. Lp02 bacteria putatively containing an inactivated *irsA*, *feoB*, or *lbtA* gene were obtained by plating the transformation mixture onto BCYE agar containing Kn. Verification of an altered *irsA* gene was done by PCR using the primer pair AL49 and AL50, confirmation of mutated *feoB* was done by PCR using the primer pair AL25 and AL26, and confirmation of mutated *lbtA* was done by PCR using the primer pair AL27 and AL28. Then, following transformation with pBSFLP, mutants harboring the desired unmarked deletion (and lacking pBSFLP) were recovered by plating onto BCYE agar containing sucrose, scored for loss of resistance to both Kn and Gm, and finally verified by PCR using the gene-specific primers. The Lp02 *irsA* mutants obtained were designated as NU476 and NU477, the Lp02 *feoB* mutants generated were named NU478 and NU479, and the Lp02 *lbtA* mutants obtained were designated as NU480 and NU481. To make Lp02 mutants lacking both *irsA* and *feoB*, we used allelic exchange to introduce the mutagenized Lp02 *feoB* allele (above) into *irsA* mutant NU476. The genotype of the *irsA feoB* mutants (designated as NU482, NU483) was verified by PCR using primers AL51 and AL52. To construct Lp02 mutants lacking both *irsA* and *lbtA*, we similarly introduced the mutagenized Lp02 *lbtA* allele into *irsA* mutant NU476. The genotype of the *irsA lbtA* mutants (NU484, NU485) was verified by PCR using primers AL27 and AL28

### Complementation analysis

Genetic complementation of the *irsA* mutants was done by reintroducing an intact copy of the *irsA* gene on a plasmid, as has been done before for complementation of various other mutants of *L. pneumophila* (14, 69). To that end, an 828 bp fragment containing the intact *irsA* ORF with its ~200 bp upstream and downstream (but no other intact gene or sRNA) was amplified from 130b DNA using primers AL63 and AL64. After the PCR product was digested with EcoRI and KpnI, the *irsA-*containing fragment was cloned into pMMBGent, a plasmid which encodes Gm resistance and is stably maintained by *L. pneumophila* (153). PCR using the vector-specific primer pair OR77 and OR78 confirmed that the newly made plasmid (i.e., p*irsA*) carried *irsA*. Plasmid p*irsA* was electroporated (149) into the 130b *irsA* mutant NU470, and transformants containing p*irsA* were obtained by plating on BCYE agar containing Gm. In a similar way, a 435 bp fragment containing the intact *irsA* ORF was amplified from Lp02 DNA using primers AL65 and AL67 and then ligated into the Cm-resistant vector pMMB2002. Finally, the resulting plasmid was electroporated into the Lp02 *irsA feoB* mutant NU482, and transformants obtained by plating on BCYE agar containing Cm.

### Intracellular infection assays

*A. castellanii* (ATCC 30234) amoebae were axenically maintained as before (154, 155). Human U937 cells (ATCC CRL-1593.2) were maintained and differentiated to a macrophage-like state using phorbol myristate acetate, as previously done (152, 156). The amoebae were inoculated with *L. pneumophila* and intracellular infection monitored, as before (14, 154). Briefly, bacteria that had been grown on BCYE agar for 3 days at 37°C were inoculated onto amoebal monolayers at a multiplicity of infection (MOI) equal to 0.1, and then 0, 24, 48, and 72 h later, aliquots taken from the co-culture supernatants were plated for CFU on BCYE agar. Differentiated U937 cells were inoculated with *L. pneumophila* strains and intracellular infection examined as before (14, 152). Briefly, bacteria that had been grown on BCYE agar for 3 days at 37°C were added into wells containing the U937 cells at a MOI = 0.5, and the bacteria centrifuged onto the monolayers. After a 2-h incubation to allow for bacterial entry, the infected wells were washed to remove remaining extracellular bacteria. At 0, 24, 48, and 72 h post-inoculation, the monolayers were lysed, and aliquots taken from the lysates were plated for CFU on BCYE agar.

### Biofilm assays

The ability of *L. pneumophila* strains to form biofilm on plastic surfaces was determined using crystal violet staining, as we and others have done before (14, 47, 115, 157). Briefly, bacteria were suspended to an OD_660_ = 0.2 in BYE or CDM broth containing differing amounts of available iron, and 200 µL aliquots of the suspensions were placed into the wells of a 96-well, polystyrene microtiter plate. After 2 days of static incubation at 30°C, 40 µL of 0.25% crystal violet was added for 15 min. After the wells were washed with ddH_2_0, the plates were allowed to air dry. Biofilm was observable as a blue ring around the walls of the inoculated wells (158). To quantitate the biofilm’s mass, 300 µL of 30% acetic acid was added into the wells to solubilize the crystal violet, and then 200 µL aliquots were transferred into another microtiter plate and read at 600 nm using a Synergy H1 plate reader (Biotek). As done before to assess *L. pneumophila* growth patterns on an agar surface (14), bacteria that had been grown for ~12 h in BYE broth were resuspended in PBS to an OD_660_ = 0.2, and then 20 µL aliquots were spotted onto BCYE agar. After 5 days of incubation at 37°C, photographic images of the bacterial growth were acquired.

### Aggregation assay

The ability of *L. pneumophila* strains to undergo auto-aggregation was assessed as before with some slight modifications (86, 87). To begin, the various bacterial strains that had been grown for 3 days on BCYE agar were suspended in 10% BYE broth/90% ddH_2_0 to an OD_660_ of ~1.0 (*n* = 3). Five-milliliter aliquots of the suspensions were then transferred into borosilicate glass tubes (13 × 100 mm) and incubated statically in an air incubator set at 30°C. Over the course of a 24-h period, bacterial sedimentation was determined by measuring drops in the OD_660_ of the suspensions and by the visual observation of pellets forming in the bottom of the tubes. The presence of *L. pneumophila* aggregates was also determined by obtaining a 5 µL aliquot of the bacterial suspension from half-way down the tube and then examining that sample under brightfield microscopy.

### *In silico* analyses

The identification of promoter regions, ORFs, and the presence of Fur boxes in promoter regions of *L. pneumophila* genes was done as before (18, 159). Predictions regarding the secreted nature of proteins encoded within the *L. pneumophila* genome were done using SignalP and PSORTb, analogously to prior examinations of secreted proteins (118, 160). Genomes and associated metadata for unique species of the orders *Berkiellales*, *Coxiellales*, *Diplorickettsiales*, *DSM-16500*, *Francisellales, Legionellales*, and *Piscirickettsiales* were identified either from the NCBI RefSeq genome database (161) or the Genome Taxonomy Database (162). An all-against-all BLAST was performed from all ORF amino acid sequences of the 154 collected genomes using DIAMOND (v.2.0.13.151) (163). The Markov Clustering Algorithm (164) was then used to identify clusters of gene families from BLASTP results (*E*-value threshold <10^−7^) using an inflation parameter value of 1.5. Amino acid sequences from gene families with greater than three homologs were aligned separately using MAFFT (v.7.407) (165) with the “--auto” parameter, and excess unaligned amino acids were trimmed using Clipkit (v.2.1.1) (166) with default parameters. GNU Parallel (167) was used to parallelize both the MAFFT alignment and Clipkit trimming steps. Gene trees were inferred from each trimmed alignment using the software package ParGenes (168), yielding maximum likelihood trees for each gene family with RAxML-NG (169). Using the gene trees inferred from ParGenes, a species tree was then estimated using the SpeciesRax (170) software package, via the GeneRax (171) executable, v.2.0.4. A predicted structure for IrsA was made using AlphaFold 3 (58) and rendered with ChimeraX software (v.1.4) (172). To discern possible relationships to known structures, we submitted the predicted structure to the DALI server (v.5) (59), as we have done before to study other bacterial proteins (93, 173).

### Statistical methods

As noted in the figure legends, each experiment utilized ≥3 technical replicates, and the values gained were given as the means and standard deviations. Unless indicated differently above, *P*-values were determined by the Student’s *t*-test (14, 93). As noted in figure legends, repeat experiments (biological replicates) were done for confirmation.

## Data Availability

The complete assembly of the strain 130b genome is under GenBank accession number GCA_041734345.1, and raw reads have been submitted to NCBI’s SRA under accession number SAMN42206255. Raw reads for RNA seq have been submitted to NCBI’s SRA under accession numbers SRR30641454-SRR30641459. The sequence for the *14915*/*irsA* gene of strain 130b has also been specifically deposited at the NCBI, i.e., BankIt2869790 irsA: PQ315881.

## SUPPLEMENTAL MATERIAL

Supplemental figures and tables spectrum.02313-24-s0001.pdf Fig S1 to S6; Tables S2 and S3.

Table S1 spectrum.02313-24-s0002.xlsx Full dataset from RNA Seq analysis.

ASM does not own the copyrights to Supplemental Material that may be linked to, or accessed through, an article. The authors have granted ASM a non-exclusive, world-wide license to publish the Supplemental Material files. Please contact the corresponding author directly for reuse.
