# Supplementary material for: Legionella pneumophila IrsA, a novel, iron-regulated exoprotein that facilitates growth in low-iron conditions and modulates biofilm formation
Source: Microbiol Spectr. 2024 Nov 29;13(1):e02313-24. doi: 10.1128/spectrum.02313-24 (PMC11705809; doi:10.1128/spectrum.02313-24)
Supplement: Supplemental figures and tables — Fig S1 to S6; Tables S2 and S3. [file spectrum.02313-24-s0001.pdf]

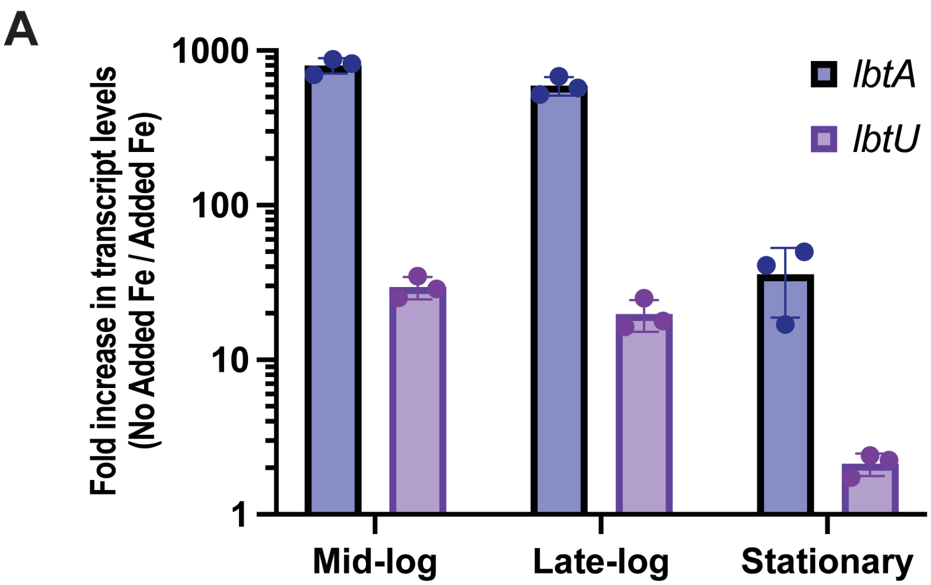

FIG S1. Effect of low-iron growth conditions on the induction of *lbtA* and *lbtU* transcription. *L. pneumophila* WT strain 130b was inoculated at an OD_{660} = 0.3 into either CDM lacking added iron (CDM -Fe) or CDM containing 5 μM ferric pyrophosphate added (CDM +Fe) and then grown to mid-log phase (OD_{660} = 0.7), late-log phase OD_{660} = 1.0), or stationary phase (OD_{660} = 1.5). Whole-cell RNA was isolated from three biological replicates (examined in triplicate), and the mRNA levels for *lbtA* and *lbtU* were determined by qRT-PCR. Presented are the means and standard deviations for the fold-increases in transcript levels when comparing samples from CDM -Fe to those from CDM +Fe.

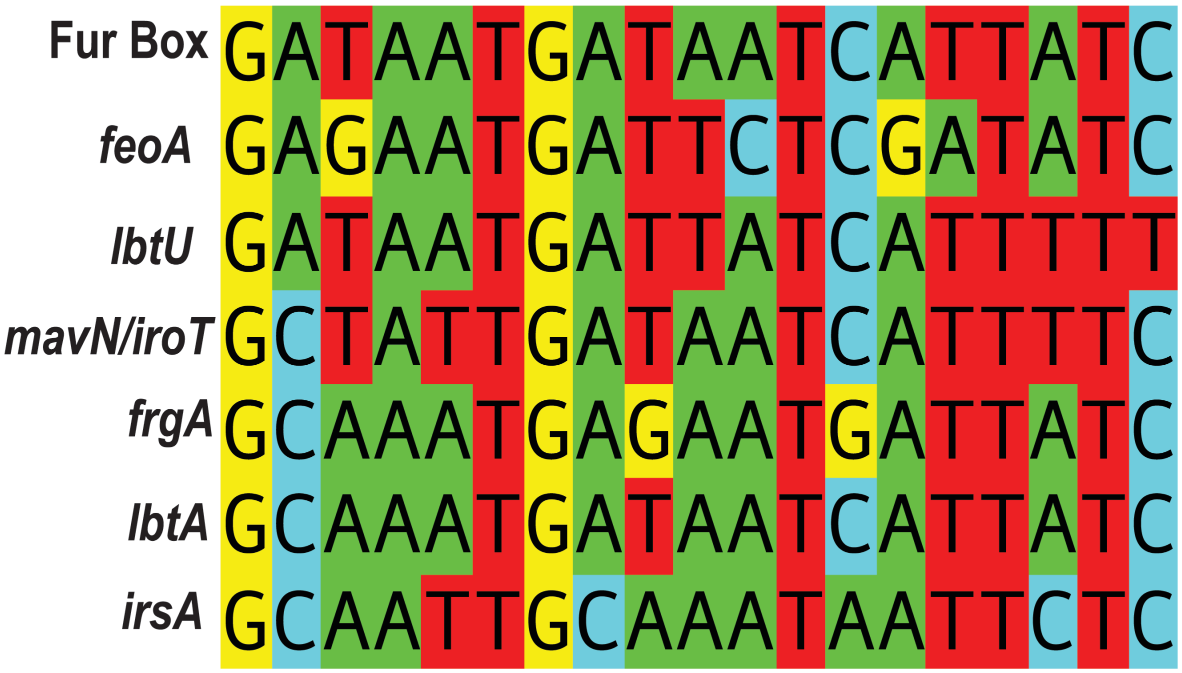

FIG S2. Putative Fur boxes upstream of the *14915 / irsA* gene and other *L. pneumophila* genes that are subject to iron-repression. A consensus Fur box appears in the top row and below that are the putative Fur boxes for *feoB, lbtU, mavN / iroT, frgA, lbtA*, and *14915 / irsA*. Conserved nucleotides are colored in the same way, with yellow for G, green for A, red for T, and blue for C.

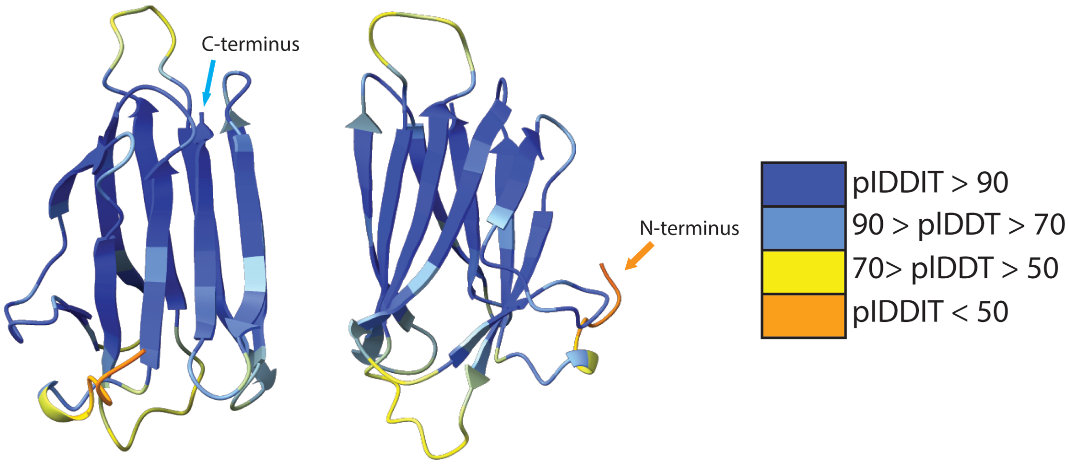

FIG S3. Model for the structure of the 14915 / IrsA protein. A predicted 3D structure for the secreted form of 14915 / IrsA (i.e., the protein without its N-terminal, 19-aa signal sequence) was determined using AlphaFold-3. Presented in two orientations (left and center), the predicted structure is color-coded in accordance with the levels of confidence (predicted local distance difference test [pLDDT] values) determined by the program (right). The N- and C-termini of the secreted protein are indicated by arrows. 14915 / IrsA is predicted to contain 10 anti-parallel beta-strands, 10 loops, and 1 immunoglobulin-like domain. Overall, the predicted template modeling (pTM) score for this protein structure was 0.86, exceeding the pTM threshold of 0.5.

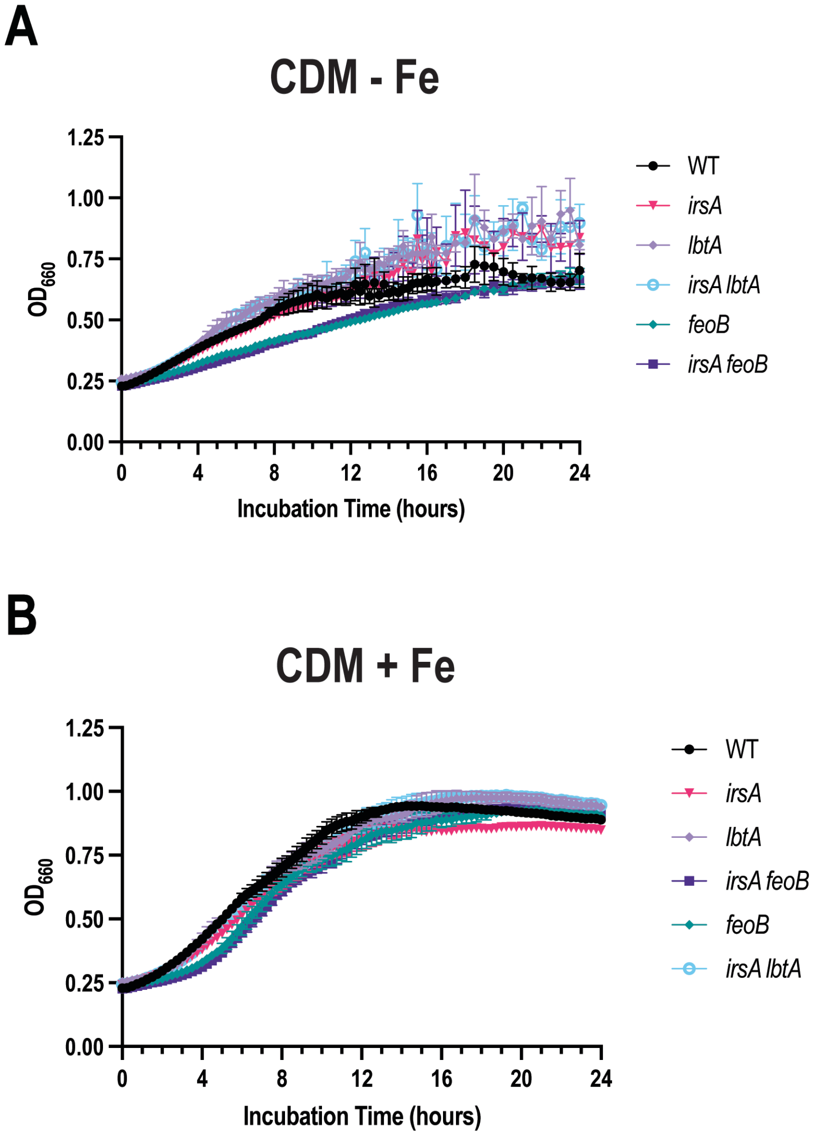

FIG S4. Growth of *irsA feoB* mutant and *irsA lbtA* mutant of strain 130b in deferrated CDM. Following overnight growth on BYE broth, WT strain 130b (WT), *irsA* mutant NU470 (*irsA*), *lbtA* mutant NU302 (*lbtA*), *irsA lbtA* mutant NU472 (*irsA lbtA*), *feoB* mutant NU458 (*feoB*), and *irsA feoB* mutant NU474 (*irsA feoB*) were placed into the wells of a 96-well microtiter plate that contained with CDM lacking any added iron (CDM – Fe) (A) or CDM containing 5 μM added ferric pyrophosphate (CDM + Fe) (B). Upon incubation at 37°C with shaking, bacterial growth was monitored by OD_{660} readings taken every h over the next 24 h. Data presented are the means and standard deviations from six technical replicates. In CDM - Fe, the *feoB* mutant and *irsA feoB* mutant grew differently from the other strains between ~ 4 h and 12 h, $P < 0.001$. The presented data are representative of the results from 3 independent experiments.

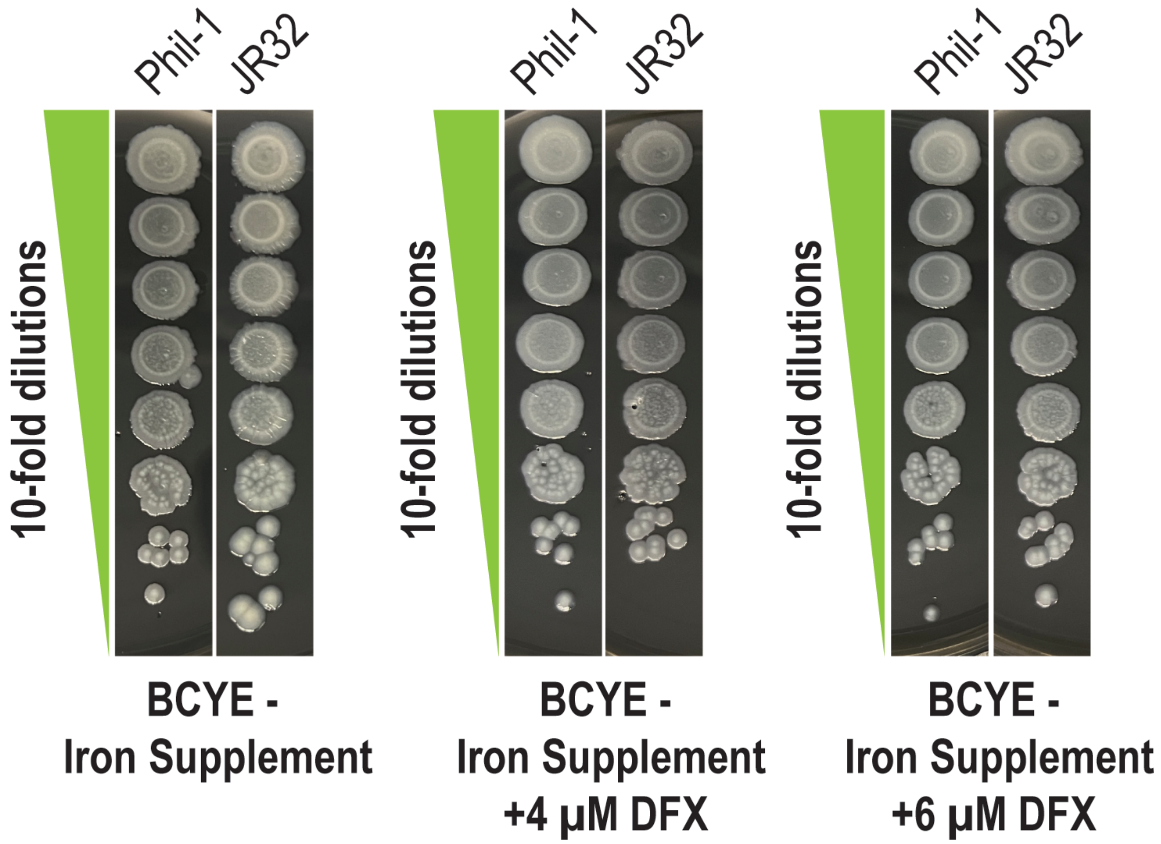

FIG S5. Relative growth of *L. pneumophila* strain JR32 on low-iron media. Following growth on thymidine-supplemented BCYE agar, WT strain Philadelphia-1 (Phil-1) and the Phil-1 derivative JR32 (JR32) were suspended in PBS to an OD_{660} = 0.3, and then 10-µl aliquots taken from a series of dilutions were spotted onto BCYE agar that either lacked the standard ferric pyrophosphate supplement (BCYE - iron supplement) or lacked the supplement and instead had increasing concentrations of the ferric iron chelator DFX added (BCYE - iron supplement +DFX). Images were taken of the areas of bacterial growth after 5 days of incubation at 37°C and are representative of results seen on two other occasions.

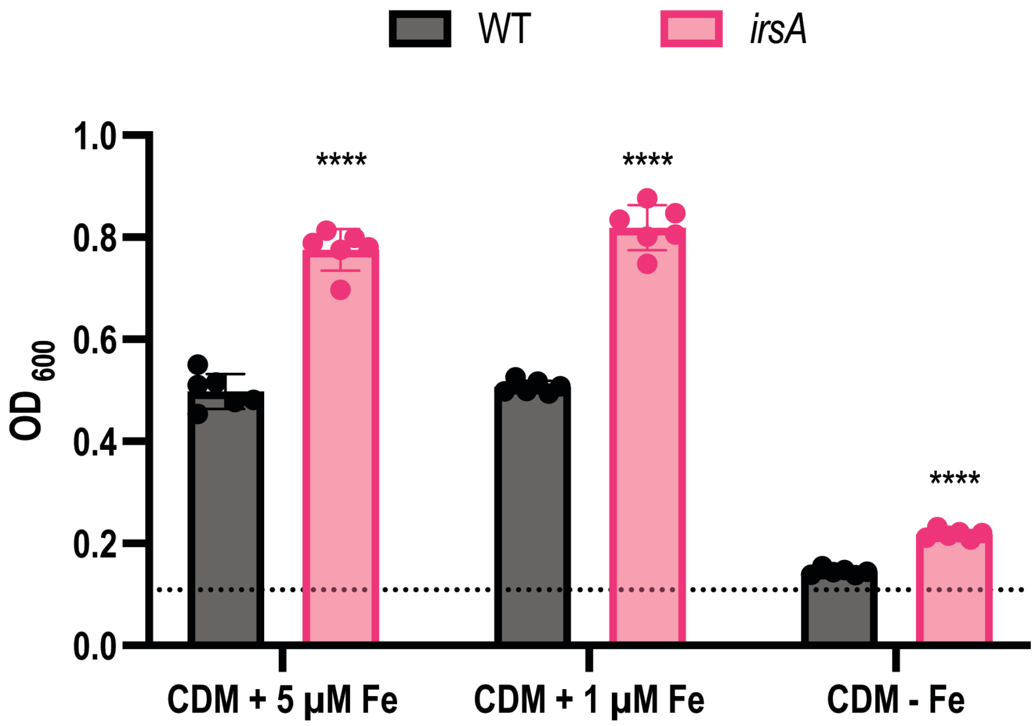

FIG S6. Further effects of *irsA* mutation on *L. pneumophila* biofilm formation. (A) Following 3 d of growth at 37°C on BCYE agar without iron supplement, WT strain 130b (WT, gray bars) and *irsA* mutant NU470 (*irsA,* pink bars) were resuspended to an OD_{660} of 0.2 in CDM broth containing either 5 µM ferric pyrophosphate added (CDM + 5 µM Fe), 1 µM ferric pyrophosphate added (CDM + 1 µM Fe), or no added iron (CDM - added Fe), and then the suspensions were added into the wells of a 96-well, plastic microtiter plate. After 3 d at 30°C, the amount of biofilm formed was determined by staining with crystal violet as read at 600 nm. The reading obtained from control wells containing only medium is indicated by the dashed horizontal line. Data presented are the means and standard deviations from six technical replicates and are representative of three independent experiments. Asterisks indicate differences in the levels of biofilm formation between WT and the mutant: ****, $P < 0.0001$.

TABLE S2. Homologs of 14915 / IrsA in other sequenced strains of *L. pneumophila*.

		% aa-identity to the corresponding proteins of strain 130b		
Strain	Source	14915 / IrsA	McoL	CorA
130b	clinical	100	100	100
Leg01/11	clinical	93.75	92.55	95
Lorraine	clinical	92.36	92.94	96.01
Paris	clinical	93.05	92.98	96.31
Mississauga	clinical	93.75	92.39	95.7
Toronto-2005	clinical	95.13	98.62	100
Alcoy	clinical	93.75	92.2	96.31
ATTC 33215	clinical	93.05	92.98	96.31
ATCC 43283	clinical	93.75	92.39	96.31
ATTC 43290	clinical	93.05	92.98	96.31
AZ00029759	clinical	99.3	99.8	100
C9_S	clinical	93.05	92.98	96.31
Corby	clinical	93.75	93.37	97.23
F4455	environmental	95.139	93.72	95.39
FFI102	clinical	96.52	97.66	99.08
FFI103	environmental	96.52	97.66	99.08
FFI329	environmental	96.52	97.66	99.08
Isolate 4368	environmental	93.75	92.2	94.17
Isolate 4788	environmental	95.13	93.37	95.39
Isolate 4825	environmental	95.13	93.37	95.39
Lens	clinical	96.51	97.61	99.08
LG51	environmental	95.93	93.37	95.39
LPE509	environmental	93.056	92.98	96.31
NMB001870	clinical	96.52	97.61	99.08
NMB001868	clinical	96.52	97.61	99.08
Philadelphia-1	clinical	93.05	92.98	96.31
SH003	environmental	95.83	93.72	95.39
Sudbury	clinical	93.05	92.98	96.31
Thunder Bay	clinical	93.05	92.98	96.31
A19030476	clinical	93.05	90.64	93.86
CL20-200363	environmental	93.75	92.35	96.19

CL20-200376	environmental	93.75	92.35	96.31
D1169	clinical	93.75	92.39	96.31
D5035	clinical	93.05	92.98	96.31
D5945	clinical	92.36	94.81	98.46
D6026	clinical	92.36	94.81	98.46
Dallas-1E	environmental	92.36	94.81	98.46
Detroit-1	clinical	92.36	94.81	98.46
GC03	environmental	95.83	99.02	100
GC04	environmental	95.83	99.02	100
GC05	environmental	95.83	99.02	100
Isolate 3969	environmental	95.83	93.37	95.39
Isolate 4343	environmental	95.83	93.37	95.39
Isolate 4809	environmental	95.13	93.37	95.39
Isolate 4826	environmental	95.83	93.37	95.39
NCTC12273	environmental	93.75	90.25	96.01
NCTC12000	clinical	93.75	92.39	96.31
Pontiac	environmental	93.05	92.15	96.31
PtVF66/2014	clinical	92.36	99.2	98.46
CL20-200126	environmental	91.67	92.53	98.46
CL20-200316	environmental	91.67	92.53	98.46
CL20-200376	environmental	93.75	92.53	96.31
D1405	clinical	92.36	91.67	98.46
D3294	clinical	91.66	91.04	98.46
D4700	clinical	93.75	92.98	98.16
SU18-MZ1	environmental	94.44	88.62	96.01
SU18-MZ2	environmental	94.44	88.62	96.01
Average % aa-identity		94.27	94.05	97.09

TABLE S3. Primers used in this study

Name	Description	Sequence (5' to 3')
AL31	lbtA qRT F	CATCGGCCTGTTGGATTAGT
AL32	lbtA qRT R	TCGTGGCATGAAATGGTAGAG
AL33	lbtU qRT F	AATCGAAGAGCGTCGCCAA
AL34	lbtU qRT R	TCGGTATTCCAAGTGCCAGAG
AL35	feoB qRT F	TTCATCAAGACAACTGGCCCGC
AL36	feoB qRT R	CAGGGTTCCACAA TGCAGAGCC
AL37	frgA qRT F	ACTCTCCCGAA TTCCAGGCCAA
AL38	frgA qRT F	TGATTCCTCCACTGCCAAGGGT
AL39	14915 qRT F	ACCTCGAAATCGGGTTCCTG
AL40	14915 qRT R	AGGCCATAGCTCAAAGGGTT
AL41	14915 _seq_F	CGCTTGGGACAATTAATCCATGCC
AL42	14915 _seq_R	CACACCCAATTATTGCTTTACTGCTTC
AL43	14915 _seq2_F	TTTGATGGTCTTTCCATGTACATGATAGTTCTATT
AL44	14915 _seq2_R	GGCTACTTTTGGGCACTTGGCTTGC
AL25	feoB_seq_F	CCAATTGTGGTAAGACAACCCTATT
AL26	feoB_seq_R	CAAGTGAAACTCCCTACTCAACTGTTG
AL45	lpg0266 5'F	GCAGCGGCCGCGTTCCGGCTTGCAATAATCTGTAG
AL46	lpg0266 5'R	GAAGCAGCTCCAGCCTACACATATTTTCCTTGTAAAAACATCAAGTCACTTGTC
AL47	lpg0266 3'F	TAAGGAGGATATTCATATCTCTATGAAGTAGCCTAAAACCACTTC
AL48	lpg0266 3'R	GGAGTCGACCAAAATCCTTACCCTATGCCAAAACATC
AL49	lpg0266_KanF	GACAAGTGACTTGATGTTTTTACAAGGAAAATATGTGTAGGCTGGAGCTGCTTC
AL50	lpg0266_KanR	GAAGTGGTTTTAGGCTACTTCATAGAGATATGAATATCCTCCTTA
AL51	lpg0266_Seq_F	GCATGAGGTATGCGATTGCAAATA
AL52	lpg0266_Seq_F	CCGGAATTAGGTTGGAAATATGGC
AL53	lpg2657 (feoB) 5'F	CGTTTAGGTCGACAACCCTTGTTAGAG
AL54	lpg2657 (feoB) 5'R	GCCTACGCGTTGATTCGCATTGGTCTGTGTAGGCTGGAGCTGCTTC
AL55	lpg2657 (feoB) 3'F	TAAGGAGGATATTCATATTTTTATCTACCGGGAAGGGGTAGTAAG
AL56	lpg2657 (feoB) 3'R	GAAGAGGTCGACAAACTGCAAATAGC
AL57	lpg2657 (feoB) KanF	GAAGCAGCTCCAGCCTACACAGACCAATGCGAATCAACGCGTAGGC
AL58	lpg2657 (feoB) KanR	CTTACTACCCCTTCCCGGTAGATAAAAATATGAATATCCTCCTTA
AL25	feoB_seq_F	CCAATTGTGGTAAGACAACCCTATT
AL26	feoB_seq_R	CAAGTGAAACTCCCTACTCAACTGTTG
AL59	lpg1325 (lbtA) 5'F	GCTAATTTATAACACCTTGC
AL18	lpg1325 (lbtA) 5'R	GAAGCAGCTCCAGCCTACACAGATTATTTCCGTGAATAAGTAAG
AL60	lpg1325 (lbtA) 3'F	TAAGGAGGATATTCATATGACTAGTTCAAAAAATGATGC
AL61	lpg1325 (lbtA) 3'R	ATAAGGCCGCAAGTACG
AL23	lpg1325 (lbtA) KanF	CTTACTTATTCACGGAAATAATCTGTGTAGGCTGGAGCTGCTTC
AL62	lpg1325 (lbtA) _ KanR	GCATCATTTTTTGAACTAGTCATATGAATATCCTCCTTA
AL27	lbtA_seq_F	GCTAATTTATAACACCTTGC
AL28	lbtA_seq_R	ATAAGGCCGCAAGTACG
AL63	irsA_EcoRI_comp_F	TGAGAATTCCAGTTAATAACGCAGTAAGCAAATCGC
AL64	irsA_KpnI_Nat_comp_R	GCAGGTACCGAAACCATGAAAGTATTAACCGCCTTCG
AL65	irsA_EcoRI_6XHIS_F	GCATGAGAATTCATGAAAAGAATTTTTTTAACATCGATGATTGGGATAGT
AL66	irsA_KpnI_6XHIS_R	TGCGGTACCTTAGTGGTGATGGTGATGATGACCGACACTAAAATTTTCCCCAACC
AL67	irsA_KpnI_comp_R	GCAGGTACCCTAACCGACACTAAAATTTTCCCCAACC
OR77	pmmBGent Vector Fwd	TCGGCTCGTATAATGTGTGG
OR77	pmmBGent Vector Rev	ACCGCTTCTGCGTTCTGATT
